# Isogenic iPSC-derived *CTBP1* mutant neuronal cells exhibit neurodevelopmental defects

**DOI:** 10.3389/fnins.2025.1695464

**Published:** 2025-12-12

**Authors:** Suhjin Lee, Selvamani Vijayalingam, Elliott Klotz, Cass Dedert, Fenglian Xu, Govindaswamy Chinnadurai, Uthayashanker R. Ezekiel

**Affiliations:** 1 Department of Health Management and Policy, College of Public Health and Social Justice, Saint Louis University, St. Louis, MO, United States; 2 Department of Genetics, Genome Engineering and Stem Cell Center (GESC@MGI), Washington University School of Medicine in St. Louis, St. Louis, MO, United States; 3 McDonnell Genome Institute, Washington University School of Medicine, St. Louis, MO, United States; 4 Department of Biology, College of Arts and Sciences, Saint Louis University, St. Louis, MO, United States; 5 Department of Molecular Microbiology and Immunology, Saint Louis University School of Medicine, Edward A. Doisy Research Center, St. Louis, MO, United States; 6 Department of Clinical Health Sciences, Doisy College of Health Science, Saint Louis University School of Medicine, St. Louis, MO, United States

**Keywords:** CtBP1, *de novo* mutation, HADDTS, transcriptional repression, isogenic cells

## Abstract

Hypotonia, ataxia, developmental delay, and tooth enamel defects syndrome (HADDTS) is a recently identified disorder linked to a heterozygous mutation in the C-terminal Binding Protein 1 (*CTBP1*) transcriptional corepressor. The predominant mutation (p.R342W) is located within the major protein binding cleft (PXDLS), crucial for CtBP1’s interaction with transcriptional regulatory proteins. To investigate the mutation’s functional consequences, we generated isogenic induced pluripotent cell lines (iPSCs) carrying the *CTBP1* mutation in heterozygous and homozygous conditions using the CRISPR/Cas9 editing method. The transcriptional profile of iPSC-derived early neurons from the isogenic wild type and *CTBP1* heterozygous and homozygous mutants was determined by genome-wide RNA sequencing. The RNA-Seq data revealed downregulation of several key transcription factors, with homozygous mutations causing more pronounced downregulation compared to heterozygous mutations. Isogenic mutant neural stem cells (NSCs) exhibited reduced adhesion and migration, along with dysregulated calcium signaling, while mutant neurons showed premature neurite outgrowth. Together, our transcriptomic and biological results provide novel insights into the role and mechanism of *CTBP1* p.R342W mutation in the defective neurodevelopmental processes.

## 1 Introduction

Early neurodevelopment encompasses vital cellular processes such as adhesion, neurogenesis, cell migration, differentiation, synaptogenesis, neuronal cell death, and synaptic rearrangement (Jiang and Nardelli, 2016). These processes must unfold in a specific spatial and temporal sequence, as any disruption can have enduring consequences on brain development (Jiang and Nardelli, 2016). This involves precise and orchestrated gene expression to regulate the fate of differentiating cells (Jiang and Nardelli, 2016). C-terminal Binding Proteins (CtBPs), which mainly function as transcriptional corepressors, have been shown to play a critical role in cell development and survival (Chinnadurai, 2002). CtBP1 plays a major role in the epithelial-mesenchymal transition (EMT), suppressing several epithelial genes, such as the E-cadherin coding gene *CDH1*, thereby causing cells to adhere less and leading to the suppression of pro-apoptotic genes (Grooteclaes et al., 2003). EMT involves cells losing their polarity and adhesion properties and transitioning to gain migratory and invasive properties (Le Bras et al., 2012). Most studies have focused on CtBP’s role in diseases, especially in several types of cancer (Chinnadurai, 2002; Filograna et al., 2024; He et al., 2021; Sahu et al., 2017). However, recent studies have been directed at elucidating CtBP’s roles in neurodevelopment and neurodegenerative diseases (Acosta-Baena et al., 2022; Hubler et al., 2012; Oma et al., 2003; Vijayalingam et al., 2020).

The CtBP family consists of two highly related paralogs—CtBP1 and CtBP2 (and their splice forms) in vertebrates (Chinnadurai, 2007). Both are highly conserved, expressed in different vertebrate tissues, and function as transcriptional corepressors (Bergman and Blaydes, 2006; Chinnadurai, 2002, 2007). CtBPs mediate transcriptional repression by targeting various chromatin-modifying factors and DNA-binding repressors in the promoter regions (Chinnadurai, 2007). CtBP1 and CtBP2 have multiple protein-protein interacting domains, including PDZ, Arg-Arg-Thr (RRT), and Pro-X-Asp-Leu-Ser (PXDLS) (Chinnadurai, 2007; Quinlan et al., 2006; Shi et al., 2003). The PXDLS-binding site/pocket is the hydrophobic cleft that interacts with other proteins that contain interacting motifs, allowing CtBP to function as a corepressor (Kuppuswamy et al., 2008; Quinlan et al., 2006).

A newly emerging syndrome, hypotonia, ataxia, developmental delay, and tooth enamel defects syndrome (HADDTS) is linked to a *de novo* heterozygous missense mutation in the *CTBP1* gene (NM_001328.2: c.1024C → T, p.Arg342Trp) (Acosta-Baena et al., 2022; Beck et al., 2016; Beck et al., 2019). Children that are heterozygous for the *CTBP1* mutation [(R343 (wild-type allele): W342 (mutant allele)] exhibit neurodevelopmental defects such as intellectual disabilities, movement disorders (ataxia and hypotonia), and cerebellar atrophy (Acosta-Baena et al., 2022; Beck et al., 2016; Beck et al., 2019; Kadhim et al., 2023; Wong et al., 2022).

The *CTBP1* mutation associated with the developmental phenotypes in HADDTS patients is located in the α-helical region (α-5), which is crucial for stabilizing the protein-protein interacting cleft (Beck et al., 2016). There are 14 reported cases of HADDTS with a heterozygous mutation in the *CTBP1* gene; 13 individuals carry the missense mutation c.991C → T [W342 (mutant allele)], and one individual carries the 2-base pair deletion c.1315_1316del CA) (Acosta-Baena et al., 2022). The two variants of the mutation are also located in the CtBP1 protein-interacting domain, PXDLS (Acosta-Baena et al., 2022). As CtBP1 associates with chromatin-modifying components such as HDAC1/2, HMTases G9a and GLA, and histone demethylase LSD1 at the PXDLS interacting domain (Shi et al., 2003), the R342W mutation may affect their recruitment to the chromatin. The 13 HADDTS patients carrying a missense mutation and the one patient with a deletion mutation suggest that the PXDLS interacting domain is critical for the function of CtBP1.

In our previous study, we generated induced pluripotent stem cells (iPSCs) from two p.R342W patients and two matching control donors. These iPSCs were differentiated into neural stem cells (NSCs) and neurons (Vijayalingam et al., 2020). Comparing the RNA-seq data of the neurons from patients and control donors revealed the downregulation of gene networks involved in neurodevelopment, synaptic adhesion, and anti-viral (interferon) responses (Vijayalingam et al., 2020). The patient-derived neurons exhibited morphological and electrophysiological abnormalities consistent with the altered gene expression patterns (Vijayalingam et al., 2020).

In this study, we generated isogenic heterozygous and homozygous mutant iPSCs harboring the missense mutation. Although a homozygous mutation has not been found in any HADDTS patients so far, possibly due to its prenatal or postnatal lethal conditions, assessing the effects of both the heterozygous and the homozygous mutations of CtBP1 can help to understand its mechanism of action. We also compared the transcriptomes of isogenic heterozygous and homozygous mutant neurons to delineate possible molecular pathways affected by *CTBP1* mutation.

Our *in vitro* studies revealed that isogenic *CTBP1* mutant NSCs exhibit significantly less adherence and migration compared to wild-type NSCs. In both mutant neurons, neurites formed prematurely, and their lengths were significantly longer than those of the wild type. Based on our transcriptome analysis of isogenic neurons, we identified downregulated transcription factors (TFs) that may be responsible for these observed phenotypes in mutant cells.

## 2 Materials and methods

### 2.1 Isogenic iPSC lines and NSC differentiation

The isogenic iPSC heterozygous (R342/W342) and homozygous mutant (W342/W342) cell lines were derived from the parental wild-type iPSC using CRISPR/Cas9 editing by the introduction of a knock-in point mutation (c.991C → T, R331W in NM_001012614.1; R342W c.1024 C → T in NM_001328.2) (Genome Engineering and Stem Cell Center@MGI, Washington University School of Medicine, Supplementary Figure 1). Validity studies, including gRNA selection, short tandem repeat (STR), off-target analysis, and karyotyping, were also conducted by the Genome Engineering and Stem Cell Center, Washington University School of Medicine (Supplementary Figures 1–3). Genome Engineering and Stem Cell Center@MGI Washington University School of Medicine confirmed successful and accurate targeted genetic modifications using next-generation sequencing. STR profiling confirmed that the parental and mutant cell lines matched at 14 of the 15 tested loci (Supplementary Figure 1). According to standard authentication criteria (American Type Culture Collection), a match of ≥ 80% indicates relatedness, and our lines showed a similarity ≥ 90% (Capes-Davis et al., 2013; Almeida et al., 2016). The single allelic difference observed at one locus is likely due to either parental mosaicism or a spontaneous allelic drift that can occur during cell culture or clonal expansion (Hameed et al., 2024).

The iPSC lines (wild type BJFF.6, heterozygous 1E11, and homozygous 1D7) were cultured in StemFlex media without antibiotics, passaged, and frozen and thawed as per the protocol (Thermo Fisher Scientific). The iPSC lines were differentiated into NSCs by plating them on Geltrex (Thermo Fisher Scientific) coated plates and growing them in PSC Neural Induction Media (NIM) as per the protocol (Thermo Fisher Scientific). The differentiated NSCs were characterized by immunocytochemistry using Nestin and Sox2, NSC markers (Supplementary Figure 4).

### 2.2 Early neuron differentiation

Early neurons were generated by differentiating NSCs. NSCs were first plated on poly L-ornithine (PLO) and laminin coated plates and grown using StemPro NSC Serum Free Media (SFM) composed of Knockout DMEM/F12, StemPro Neural Supplement, recombinant FGF-Basic (Human), recombinant EGF (Human), and GlutaMAX-I (Thermo Fisher Scientific) for the first 2 days. After 2 days, the media was replaced with and grown in Neuron Differentiation Medium (NDM) composed of 1X Neurobasal Medium, serum-free B-27 supplement, and GlutaMAX-I supplement (Thermo Fisher Scientific). The 14-day differentiated neurons were characterized using immunocytochemistry for the neuron-specific cytoskeletal marker beta-3 tubulin (TUJ1).

### 2.3 Imaging and Immunocytochemistry

For neurospheres, neurite measurements, and the wound healing/scratch assay, images were taken on an inverted microscope (Leica Microscope DMIL with attached camera). For immunocytochemistry, cells or neurospheres were fixed for 30 min with 4% paraformaldehyde and subsequently washed 3 times with 1X PBS, permeabilized for 5 min with 0.3% Triton in 1X PBS, and blocked with 5% goat serum diluted in 1X PBS for 1 h. The samples were then incubated overnight with mouse monoclonal anti-beta-3 tubulin (1:100, Sigma, Cat. No. T0198) or rabbit monoclonal anti-microtubule-associated protein 2 (MAP2) antibodies (1:200, Cell Signaling technology, Cat. No. 8707). The next day, cells were washed three times with 1X PBS. Cells were then incubated with either Alexa Fluor 488 goat anti-mouse IgG secondary antibody (1:100, Thermo Fisher Scientific, Cat. No. A11029) for labeling beta-3 tubulin or Alexa Fluor 488 goat anti-rabbit IgG secondary antibody (1:100, Cell Signaling Technology, Cat. No. 4412) for labeling MAP2 for 1 h at room temperature (21–22°C) in the dark. Cells were rinsed three times with 1X PBS and mounted using Fluroshield with 4’,6-diamidino-2-phenylindole (DAPI) (1:1,000, Sigma, Cat. No. F6057). Samples were acquired and viewed using laser scanning confocal microscopy (Leica TCS SP8 STED 3X super-resolution system) under a 40X objective at 488 nm excitation (green, beta-3 Tubulin and MAP2) with a 590/50 emission filter. Stack images of 0.7 μm were first collected and compressed into single 3D images. Image acquisition parameters for wild-type and mutant neurons were kept the same. Cytation3 Cell Imaging Multi-Mode Reader (BioTek) was used to acquire images of neurospheres at 4X magnification.

### 2.4 RNA sequencing and analysis

For the transcriptional profiling study, we isolated RNA from 14-day differentiated neurons from wild-type and mutant NSCs (section 2.2). Three biological replicates were used for each cell line that were from three different 60 mm dishes. Cells were lysed with Trizol (Zymo Research, Irvine, CA), and total RNA purification was conducted using the Direct-Zol RNA kit (Zymo Research, Irvine, CA) following the manufacturer’s protocol. RNA concentration was determined by the NanoDrop spectrophotometer.

Total RNA integrity was determined using Agilent Bioanalyzer or 4200 TapeStation. Library preparation was performed with 500 ng to 1 μg of total RNA. Ribosomal RNA was blocked using FastSelect reagents (Qiagen) during cDNA synthesis. RNA was fragmented in reverse transcriptase buffer with FastSelect reagent and heating to 94 °C for 5 min, 75 °C for 2 min, 70 °C for 2 min, 65 °C for 2 min, 60 °C for 2 min, 55 °C for 2 min, 37 °C for 5 min, 25 °C for 5 min. mRNA was reverse transcribed to yield cDNA using SuperScript III RT enzyme (Life Technologies, per manufacturer’s instructions) and random hexamers. A second strand reaction was performed to yield ds-cDNA. cDNA was blunt ended, had an A base added to the 3’ ends, and then had Illumina sequencing adapters ligated to the ends. Ligated fragments were then amplified for 15 cycles using primers incorporating unique dual index tags.

The cells were indexed, pooled, and sequenced on an Illumina NovaSeq 6000. Base calls and demultiplexing were performed with Illumina’s bcl2fastq software and a custom python demultiplexing program with a maximum of one mismatch in the indexing read. RNA-seq reads were then aligned to the Ensembl release 76 primary assembly with STAR version 2.5.1a1. Gene counts were derived from the number of uniquely aligned unambiguous reads by Subread:featureCount version 1.4.6-p52. Isoform expression of known Ensembl transcripts were estimated with Salmon version 0.8.23. Sequencing performance was assessed for the total number of aligned reads, total number of uniquely aligned reads, and features detected. The ribosomal fraction, known junction saturation, and read distribution over known gene models were quantified with RSeQC version 2.6.24.

All gene counts were then imported into the R/Bioconductor package EdgeR5, and TMM normalization size factors were calculated to adjust for samples for differences in library size. Ribosomal genes and genes not expressed in the smallest group size minus one sample greater than one count-per-million were excluded from further analysis. The TMM size factors and the matrix of counts were then imported into the R/Bioconductor package Limma6. Weighted likelihoods based on the observed mean-variance relationship of every gene and sample were then calculated for all samples with the voomWithQualityWeights7. The performance of all genes was assessed with plots of the residual standard deviation of every gene to their average log-count with a robustly fitted trend line of the residuals. Differential expression analysis was then performed to analyze differences between conditions, and the results were filtered for only those genes with Benjamini-Hochberg false-discovery rate adjusted *p* = 0.05.

To find the most critical genes, the raw counts were variance stabilized with the R/Bioconductor package DESeq211 and were then analyzed via weighted gene correlation network analysis with the R/Bioconductor package WGCNA12. Briefly, all genes were correlated across each other by Pearson correlations and clustered by expression similarity into unsigned modules using a power threshold empirically determined from the data. An eigengene was then created for each *de novo* cluster and its expression profile was then correlated across all coefficients of the model matrix. Because these clusters of genes were created by expression profile rather than known functional similarity, the clustered modules were given the names of random colors where gray is the only module that has any pre-existing definition of containing genes that do not cluster well with others.

To identify transcriptional variation among the three *CTBP1* iPSC lines, three-dimensional principal component analysis (PCA) was performed using normalized RNA-seq expression data (Supplementary Figure 5). The first three principal components (PC1, PC2, and PC3) together captured the majority of total variance, with PC1 and PC2 accounting for the largest portion, followed by PC3. The PCA plot demonstrates distinct clustering of the BJFF.6 wild-type, 1E11 heterozygous, and 1D7 homozygous *CTBP1* R342W iPSC lines, indicating clear genotype-specific transcriptional differences. Replicates within each group clustered tightly together, reflecting high consistency within each cell line and minimal technical variation. These results confirm that CRISPR/Cas9 editing and subsequent clonal derivation did not introduce broad transcriptional artifacts, and that the observed differences primarily reflect the intended genetic modification.

To visualize transcriptomic differences among the *CTBP1* iPSC lines, a heat map was generated using the set of differentially expressed genes identified by pairwise comparisons (Supplementary Figure 5). The heat map shows distinct clustering of the BJFF.6 (wild type), 1E11 (heterozygous), and 1D7 (homozygous) *CTBP1* R342W iPSC lines, indicating clear genotype-dependent transcriptional profiles. Replicates within each cell line grouped closely together, demonstrating high consistency within each cell line and reproducibility of gene expression patterns. These results support the PCA findings and confirm that transcriptional variation is primarily driven by the *CTBP1* genotype rather than technical variability.

### 2.5 Cell adhesion assay

A “flipping” assay was used to determine the adherence activity of control and *CTBP1* mutant NSCs (Langhe et al., 2016). Cells were counted and plated (1 X 10^4^ cells per well) onto two 96-well black/transparent bottom plates and incubated for 30 min at 37*^o^*C in Hank’s Balanced Salt Solution (HBSS with Ca^++^, Mg^++^, no phenol red). One of the two plates was flipped over and shaken to remove nonadherent cells. Both plates were incubated with 100 μL of 1 μm Calcein AM (Thermo Fisher Scientific) in HBSS for 30 min at 37*^o^*C. Cytation3 Cell Imaging Multi-Mode Reader (BioTek) was used to measure the Calcein AM signal (Ex/Em 485/530 nm). Calcein AM is cleaved by the intracellular esterase enzyme and its cleaved product produces highly fluorescent molecules. The percentage of adhered (flipped) cells was calculated based on the total un-flipped cells. Three independent experiments were performed. For each experiment, the averages of four replicate wells (in each plate) were used for each cell line. Comparisons between control and mutant adherence were performed using two-way ANOVA with Tukey’s post-hoc test. All experimental data were reported as mean ± SEM. *P* <0.05 was considered statistically significant.

### 2.6 Wound healing/scratch assay

NSCs were grown to 100% confluency in a 6-well plate, two wells being used for each cell line. A vertical line was scratched from top to bottom in the monolayer using a p200 pipette tip, cells were washed, and fresh NIM was added. Cell migration in the scratched area was observed by photographically assessed at 0, 24, and 48 h after scratching. The area of closure was measured from images acquired using ImageJ software.^1^ Three independent experiments were performed. For each experiment, the averages of the duplicate wells were used for each time point. Statistical analysis was performed using one-way ANOVA with Tukey’s post-hoc test. *P* < 0.001 was considered statistically significant.

Percent closure was calculated using the following formula:


$$\mathrm{Percent}\,\mathrm{closure}=\left(\frac{\mathrm{Area}\,\mathrm{of}\,\mathrm{original}\,\mathrm{wound}-\mathrm{Area}\,\mathrm{of}\,\mathrm{wound}\,\mathrm{during}\,\mathrm{healing}}{\mathrm{Area}\,\mathrm{of}\,\mathrm{original}\,\mathrm{wound}}\right)*100$$

### 2.7 Proliferation assay

For each experiment, three Geltrex-coated 96-well cell culture plates were used. For each cell line, 5,000 NSCs were plated per well in triplicates and only media was added to three wells for a blank reading. The cells were incubated overnight in the incubator (37°C, 5% CO_2_). At 24, 48, and 72 h after the initial seeding of cells, each plate for the corresponding time point was retrieved from the incubator for analysis. The cell culture medium (NIM) of the removed plate was aspirated, washed twice with DPBS with calcium and magnesium. A working stock solution concentration of 1 μm Calcein AM dye in HBSS was added to each well and the cells were incubated for 30 min. The fluorescence was then measured in Cytation3 Cell Imaging Multi-Mode Reader (BioTek) at an excitation wavelength set at 485 nm and emission wavelength at 530 nm. The fluorescence measured at each time point corresponds to the number of cells in the well. The fluorescence of the blank wells was subtracted from the wells with the cells to calculate relative fluorescence. Three independent experiments were performed with the averages of the triplicates per cell line taken from each experiment. Statistical analysis was performed using one-way ANOVA, and no significant differences between the cell lines were found.

### 2.8 Neurosphere migration assay

NSCs were detached from the plate with StemPro Accutase (Thermo Fisher Scientific), centrifuged, and suspended in NSC SFM. 5,000 cells (100μL) were added to each well of a 96-well low adherence U-bottom plate (Biofloat, Sarstedt). After 48 h, images of each neurosphere were taken. To ensure that the neurospheres are similarly sized between cell lines in each experiment, ImageJ was used to measure the diameter of each cell line. The neurosphere diameter of the mutants were normalized to the wild-type neurospheres and the fold-change values were calculated. Three independent experiments were conducted. For each experiment, the average of the minimum of five neurospheres from separate wells were taken for each cell line. Statistical analysis was performed using one-way ANOVA. No significant differences in neurosphere diameter were observed between cell lines within each experiment.

The neurospheres were then transferred from the U-bottom plate using wide-bore p200 pipette tips to the center of each well of the 24-well plate pre-coated with PLO and laminin. The neurospheres were grown in NDM. After the neurospheres were attached to the plate and differentiated for 5 days, neurospheres were fixed and stained with DAPI and MAP2 antibodies to detect neurons and cell migration, respectively.

### 2.9 Neurite measurement

Neurons were differentiated (section 2.2), and the neurites were measured on day 7 of differentiation. The NeuronJ plugin of ImageJ software (see text footnote 1) was used to measure the neurite length and calculate the number of cells. Using the neurite measurement data, we calculated the cells with neurites/total cells, average neurite length, and number of neurites per cell. Each cell body with traced neurites were classified as “cells with neurites.” The neurites were semi-automatically traced using segmented lines, which were then measured to find the average neurite length. The number of neurites extending from each cell that contained the cell body was measured to calculate the number of neurites/cell. Four independent experiments were performed. In each experiment, each cell line was cultured in three wells, and one image was taken from a randomly selected area in each well. Averages of the three wells were taken. Statistical analysis was performed using one-way ANOVA with Tukey’s post-hoc test.

### 2.10 Calcium imaging

Calcium (Ca^2+^) influx was measured using a Fura 2-AM ester (Invitrogen, Cat. N. F1221), which binds selectively to Ca^2+^. The cells were washed twice with HBSS, then incubated in HBSS containing 5 μM Fura 2-AM at 37 °C for 30 min. After incubation, the cells were washed four times with HBSS before imaging. Fura-2 was excited at its excitation maxima (340 nm and 380 nm) sequentially *via* a Lambda XL instrument equipped with a high-speed wavelength switcher (Sutter Instrument), then imaged with a microscope equipped with a Retiga R1 camera (QImaging). MetaFluor imaging software (version 7.8.2.0, Molecular Devices, LLC) was used to acquire fluorescence intensity, with a higher ratio of 340 nm to 380 nm indicating greater Ca^2+^ influx. The background was subtracted from fluorescence intensity values for cells using an empty region of interest as a negative control. The degree of Ca^2+^ influx was quantified as the maximum increase from the mean baseline 340/380 nm ratio (peak Δ 340/380 nm ratio). Baseline Ca^2+^ levels were determined from recording intervals in which no Ca^2+^ spiking activity was observed.

Calcium imaging data were quantified at the single-cell level. CtBP1-mutant NSCs exhibited reduced adhesion and survival, which limited the number of independently plated coverslips obtainable per preparation, especially for Ca^2+^ imaging experiments requiring multiple washes of Ca^2+^ dye after incubation and before imaging. Across the study, we analyzed at least 50 NSCs derived from two to three independent culture preparations (biological replicates). From each preparation, multiple coverslips and multiple fields of view were imaged and cells were randomly selected from different fields to minimize sampling bias. Each cell’s calcium response (basal calcium, spiking frequency, and Δ peak amplitude) was quantified individually and pooled for statistical comparisons. The single-cell analytical approach follows previously published studies in calcium imaging, neurite outgrowth, and electrophysiology when cell yield is constrained and cell-to-cell heterogeneity is biologically meaningful (Chiu et al., 2018; Morita et al., 2003; Kikuchi et al., 2022).

Among wild-type cells, 20 out of 72 exhibited no spike events during the recording period; to ensure fair comparisons among neurons displaying spontaneous Ca^2+^ transients, these cells were excluded from the analysis, resulting in 52 cells included in the wild-type group. All 72 cells from the mutant groups exhibited at least one Ca^2+^ spike event during the recording period and were included in the analysis. 72 cells were imaged from different dishes, each of which constituted a different experimental replicate. The wild-type and homozygous cell lines had three replicates, while the heterozygous group had two replicates.

Parametric tests (one-way ANOVA followed by Tukey’s post-hoc test) were used when assumptions of normality were met. To account for potential non-normality, nonparametric analyses were also performed using Kruskal–Wallis tests followed by Dunn’s multiple comparisons, which yielded consistent statistical outcomes, supporting the robustness of the observed group differences.

### 2.11 Statistical analysis

Analyses and graphical representations were performed using GraphPad Prism 9.5.1 software (GraphPad Software Inc.). Statistical analysis was performed using one-way ANOVA with Tukey’s post-hoc test for the wound healing/scratch assay, proliferation assay, neurosphere migration assay, neurosphere diameter measurement, neurite measurement, and calcium imaging. Statistical analysis was performed using two-way ANOVA with Tukey’s post-hoc test for the cell adhesion assay. As mentioned in section 2.10, to support parametric analysis, nonparametric analyses of Kruskal-Wallis test followed by Dunn’s multiple comparison test were also conducted for calcium imaging. Differences were considered statistically significant when the *p*-value was less than 0.05 (*p < 0.05, **p < 0.01, ***p < 0.001, and ****p < 0.0001).

## 3 Results

### 3.1 Neuronal cell models

To explore how the *CTBP1* mutation affects genome-wide gene expression while avoiding variability and confounding factors, such as different genetic backgrounds, pathological severity, and epigenetic changes associated with environmental factors, we generated isogenic iPSC lines (Kawatani et al., 2021). In a previous study (Vijayalingam et al., 2020), we generated iPSCs from two patients harboring the *CTBP1* R342W mutation and from two age-matched controls to understand the pathophysiology of HADDTS patients. Early neurons were derived from iPSCs, and a comparison of RNA-seq data from patients and normal donors revealed the downregulation of several genes that are involved in neurodevelopment and synaptic adhesion (Vijayalingam et al., 2020).

**FIGURE 1:**
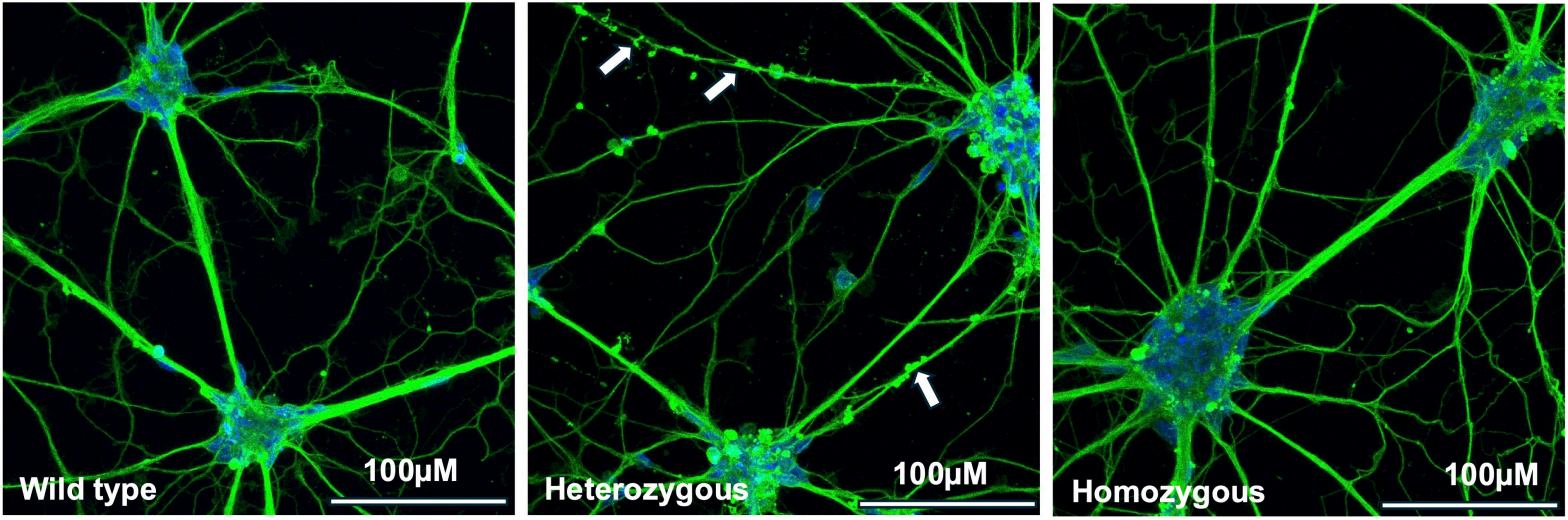
Immunofluorescence analysis of isogenic wild-type and *CTBP1* mutant neurons. Isogenic wild-type (BJFF.6) and *CTBP1* heterozygous and homozygous mutant neurons differentiated from iPSC-derived NSCs. Neurons were stained with beta-3 tubulin (green) antibodies and DAPI (blue) on day 14 of differentiation. The arrows in the heterozygous panel indicate bead-like structures (puncta) observed in the neurites. Scale bar: 100 μm (40×).

Isogenic cell lines with a knock-in point mutation (c.991C → T, R331W in NM_001012614.1; R342W c.1024 C → T in NM_001328.2) for *CTBP1* were generated from the parental iPSC line BJFF.6 using CRISPR/Cas9 genome editing (Supplementary Figure 1). The parental iPSC line (wild type) and the isogenic iPSC lines with the *CTBP1* heterozygous and homozygous mutant iPSCs were used to generate NSCs, which were then differentiated into 14-day differentiated early neurons. Neurons were stained with beta-3 tubulin (green), a specific marker for neuronal identity that stains the cell bodies and neurites, to visualize the morphological differences between wild-type and mutant neurons. DAPI (blue) staining localized the nuclei of the neurons. We observed morphological differences in the neurites of heterozygous neurons, including puncta on the neurites (Figure 1). The discussion section consists of our possible explanation for the presence of puncta and why it is not observed in the homozygous mutants.

We performed genome-wide transcriptome analysis of differentiated early neurons of wild-type and *CTBP1* mutant cells using RNA-Seq. To understand how the *CTBP1* mutation affects some of the neurodevelopmental processes, we performed *in vitro* assays using NSCs and neurons.

### 3.2 Transcriptomic profiling of isogenic NSC-derived neurons

Total RNA was isolated from 14-day differentiated neurons for transcriptomic profiling studies. We prepared RNA from wild-type, *CTBP1* heterozygous, and *CTBP1* homozygous neurons. The cDNA generated from RNA was sequenced on an Illumina Novaseq6000.

**FIGURE 2:**
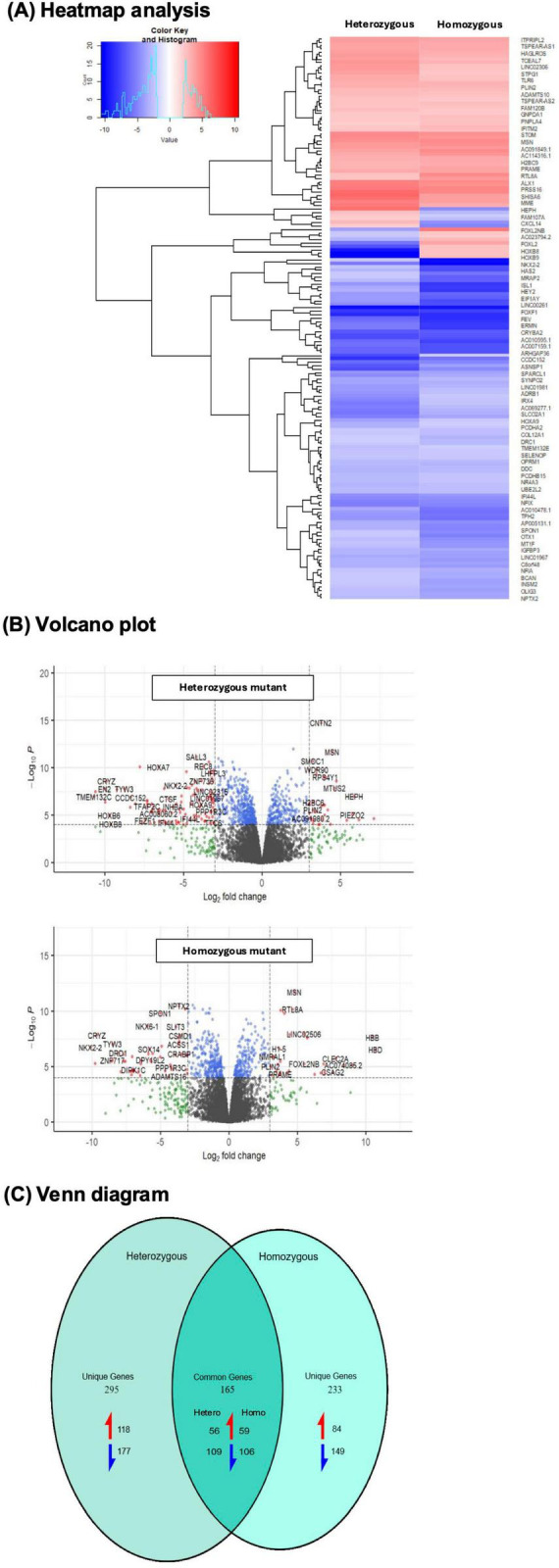
Heatmap, volcano plot, and Venn diagram of differentially expressed genes. **(A)** Heatmap analysis. Heatmap generated from significantly differentially expressed genes of all biotypes in heterozygous and homozygous isogenic neurons compared to wild-type isogenic neurons. Gene significance was calculated using adjusted *P* < 0.05 and log 2-fold changes ± 2. Red color represents upregulated and blue color represents down regulated transcripts. Most of the genes are downregulated in the *CTBP1* isogenic mutant neurons. **(B)** Volcano plot. Differentially expressed genes between isogenic wild-type and mutant neurons shown in a volcano plot. Significantly downregulated (blue) transcripts have a log fold-change < -2 and an adjusted *p* < 0.05. Significantly upregulated (red) transcripts have a log fold-change > 2 and adjusted *p* < 0.05. Volcano plots of 18,776 gene transcripts showed differential expression between mutant and wild-type neurons. Among these, 14,157 were protein-coding transcripts, and 2,884 were LncRNA. Out of the protein-coding transcripts, 210 were downregulated, and 149 were upregulated in heterozygous mutants. Out of the protein-coding transcripts, 202 were downregulated, and 106 were upregulated in homozygous mutants. **(C)** Venn diagram. Unique genes that include all biotypes present in heterozygous and homozygous and common genes between the isogenic mutants represented in a Venn diagram. Genes which were significantly differentially expressed are calculated using LogFC ± 2 and adjusted *p* < 0.05. There were 295 uniquely expressed transcripts in heterozygous isogenic neurons, of which 118 were upregulated (red arrow), and 177 (blue arrow) were downregulated compared to wild-type isogenic neurons. In the homozygous neurons, 233 unique transcripts were identified, of which 84 were upregulated and 149 were downregulated, compared to wild-type isogenic neurons. There were 165 transcripts commonly expressed between both mutants, of which 56 and 59 transcripts were upregulated, and 109 and 106 were downregulated in heterozygous and homozygous neurons, respectively.

Our analysis revealed 18,776 common gene transcripts between the heterozygous, homozygous, and wild-type groups. Among these, 14,157 were protein-coding transcripts and 2,884 were lncRNA transcripts. After differential expression analysis, we found 186 significantly downregulated (log 2-fold change ≤ 2) and 174 significantly upregulated (log 2-fold change ≥ 2) protein-coding transcripts that were deemed statistically significant (*p* < 0.05) in the heterozygous mutants relative to the wild type. For the homozygous mutants relative to the wild type, we found 255 significantly downregulated and 143 significantly upregulated protein-coding transcripts that were deemed statistically significant (*p* < 0.05). Overlapping differentially expressed genes of both mutants are shown in Figure 2A.

The majority of the genes in *CTBP1* heterozygous and homozygous mutants were downregulated when compared to wild-type neurons (Figure 2A). The volcano plot shows that significantly downregulated (blue) transcripts have a log fold-change ≤ -2 and an adjusted *p* < 0.05, while upregulated (red) transcripts have a log fold-change ≥ 2 and an adjusted *p* < 0.05 (Figure 2B).

We hypothesized that in the *CTBP1* homozygous mutant, the genes downregulated would be several-fold lower compared to heterozygous mutants. Therefore, we identified the commonly expressed genes in homozygous and heterozygous mutants based on the LogFC ± 2 and adjusted *p* < 0.05 between the mutants and wild type. The commonly expressed genes and unique genes in each mutant are shown in the Venn diagram (Figure 2C). There were 295 uniquely expressed transcripts in the *CTBP1* heterozygous mutant neurons, of which 118 were upregulated (red arrow) and 177 (blue arrow) were downregulated compared to wild-type neurons. In the *CTBP1* homozygous mutant neurons, 233 unique transcripts were identified, of which 84 were upregulated and 149 were downregulated when compared to wild-type neurons (Figure 2C).

There were 165 transcripts commonly expressed in both mutants, of which 56 and 59 transcripts were upregulated, and 109 and 106 were downregulated in heterozygous and homozygous mutant neurons, respectively (Figure 2C; Supplementary Table 1). We compared commonly expressed genes that were highly downregulated in homozygous mutants to the heterozygous mutants by dividing the homozygous logFC by the heterozygous logFC value. We found 26 genes that had > 1.5-fold downregulation in homozygous compared to heterozygous mutants (Supplementary Table 2), of which three were non-coding lncRNA, 10 were TFs, and one was a transcription repressor, *INSM2*. Sonic Hedgehog (Shh), a morphogen which is critical for neurodevelopment, also had > 1.5-fold downregulation. There were no TFs with > 1.5-fold upregulation in homozygous compared to heterozygous mutants. Table 1 shows the transcriptional regulator genes and their fold downregulation in homozygous compared to heterozygous mutant neurons. The functional significance of these TFs’ roles in neurodevelopment is explained in the discussion section.

**TABLE 1:** Transcription factors with > 1.5-fold logFC downregulation in homozygous compared to heterozygous mutants.

Gene name	logFC hetero	Adj. *P*-value hetero	logFC homo	Adj. *P*-value homo	Homo logFc/hetero logFC	Gene function	References
GATA3	-2.14	7.90E–09	-9.77	5.38E–06	4.565421	Cell migration, neuronal differentiation, and neural activity in adult retinal neurocyte	(Chen et al., 2022)
HEY2	-3.37	1.89E–05	-8.24	3.84E–03	2.445104	Maintenance of neural cells, prevents premature neuronal differentiation	(Fujitani et al., 2010)
BEST3	-3.4	3.14E–04	-7.85	1.18E–04	2.308824	Encodes calcium activated chloride channel that controls intracellular calcium release	(Matchkov et al., 2008)
OTX1	-2.3	9.39E–04	-5.13	2.54E–04	2.230435	Proliferation and differentiation of cortical progenitors	(Huang et al., 2018)
ISL1	-4.32	1.12E-05	-8.09	1.20E-04	1.872685	Plays crucial role in early neuron development, including proliferation and differentiation.	(Zhang et al., 2018)
NKX2-2	-5.47	7.03E—09	–10.1	1.78E–07	1.846435	Proliferation of neural progenitors, neuronal differentiation, ventral neuronal patterning during development, role in graded shh, and forming neuronal identity	(McMahon, 2000)
SP9	-2.16	5.61E-03	-3.88	1.37E-03	1.796296	Interneuron development, tangential migration, and spiny neuron production.	(Li et al., 2018)
BARHL1	-2.7	4.61E–02	-4.66	2.69E–02	1.725926	Migration and survival of cerebellar granule cells	(Li et al., 2004)
ARX	-2.68	2.89E-03	-4.39	2.07E–03	1.63806	Migration and communication of neurons in developing brain.	(Lim et al., 2024)
OLIG3	-2.55	8.13E-03	-3.93	9.92E-03	1.541176	Development of the earliest rhombic lip and cerebellar derivatives	(Lowenstein et al., 2021)

Commonly expressed genes in homozygous and heterozygous mutants were identified based on the LogFC ± 2 and adj. *p*-value < 0.05 when compared to wild type. Gene names and their corresponding functions, based on published literature, are also included in the table.

Several genes were found to be downregulated in heterozygous cells, suggesting that the pathogenic *CTBP1* allele may contribute to a dominant negative effect. The corepressor function of CtBP1 is mediated by its dimeric form (Chinnadurai, 2007). The pathogenic allele codes for the mutant monomeric protein (mt) and the wild-type allele codes for the wild-type monomer (wt). In heterozygous cells, different combinations of CtBP1 monomers can result in three different types of homodimeric proteins – wt/wt, wt/mt, and mt/mt.

The association of mutant monomer(s) forming homodimers (wt/mt and mt/mt) can lead to an abnormal repressor complex that interferes with CtBP1’s normal function. More specifically, the mutant monomer may impair the function of the wild-type monomer by “poisoning” the dimer, preventing proper dissociation from the target gene promoter and causing sustained repression of gene expression. Indeed, we found that the majority of the transcripts are downregulated based on our transcriptome data (Supplementary Table 1).

We also found that a few transcripts are oppositely upregulated or downregulated between the homozygous and heterozygous mutants (Supplementary Table 1). Nine genes were downregulated in heterozygous *CTBP1* mutant neurons, whereas they were upregulated in homozygous mutant neurons (highlighted in green in Supplementary Table 1). Additionally, six were upregulated in heterozygous mutant neurons, whereas they were downregulated in homozygous mutant neurons (highlighted in yellow in Supplementary Table 1). We propose that the function of a wt/mt homodimer vs. a wt/wt homodimer could lead to the opposite upregulation and downregulation seen in heterozygous mutants. A CtBP1 monomer can also potentially associate with a CtBP2 monomer, forming a heterodimer which also functions as a transcriptional corepressor. Therefore, a heterodimer containing a mutant protein in heterozygous and homozygous mutants can also result in different gene expression outcomes. The possible mechanism is explained in the discussion section.

Functional Gene Ontology (GO) analysis reveals that the *CTBP1* mutants significantly impact both biological processes and cellular components associated with neurodevelopment. Specifically, pathways involved in nervous system development and system development showed the highest number of downregulated genes with a fold enrichment greater than 1.5-fold (Supplementary Figure 6). In terms of cellular components, both mutants were impaired in cell junction and synapse function, as well as in axon and neuron projection. Based on the GO analysis, CtBP1 affects various neurodevelopmental functions (Supplementary Figure 6).

### 3.3 Physiological and biological activities of isogenic heterozygous and homozygous *CTBP1* p.R342W-mutated NSCs and neurons

To evaluate the effect of the *CTBP1* mutation on adhesion, migration, and calcium (Ca^2+^) transients, assays were performed using wild-type and mutant NSCs. To understand the effect of *CTBP1* mutation on early neurons, 7-day differentiated early neurons were assessed for neurite outgrowth, and a 3-dimensional neurosphere assay was performed to assess the migration of 5-day differentiated early neurons.

#### 3.3.1 Adhesion

**FIGURE 3:**
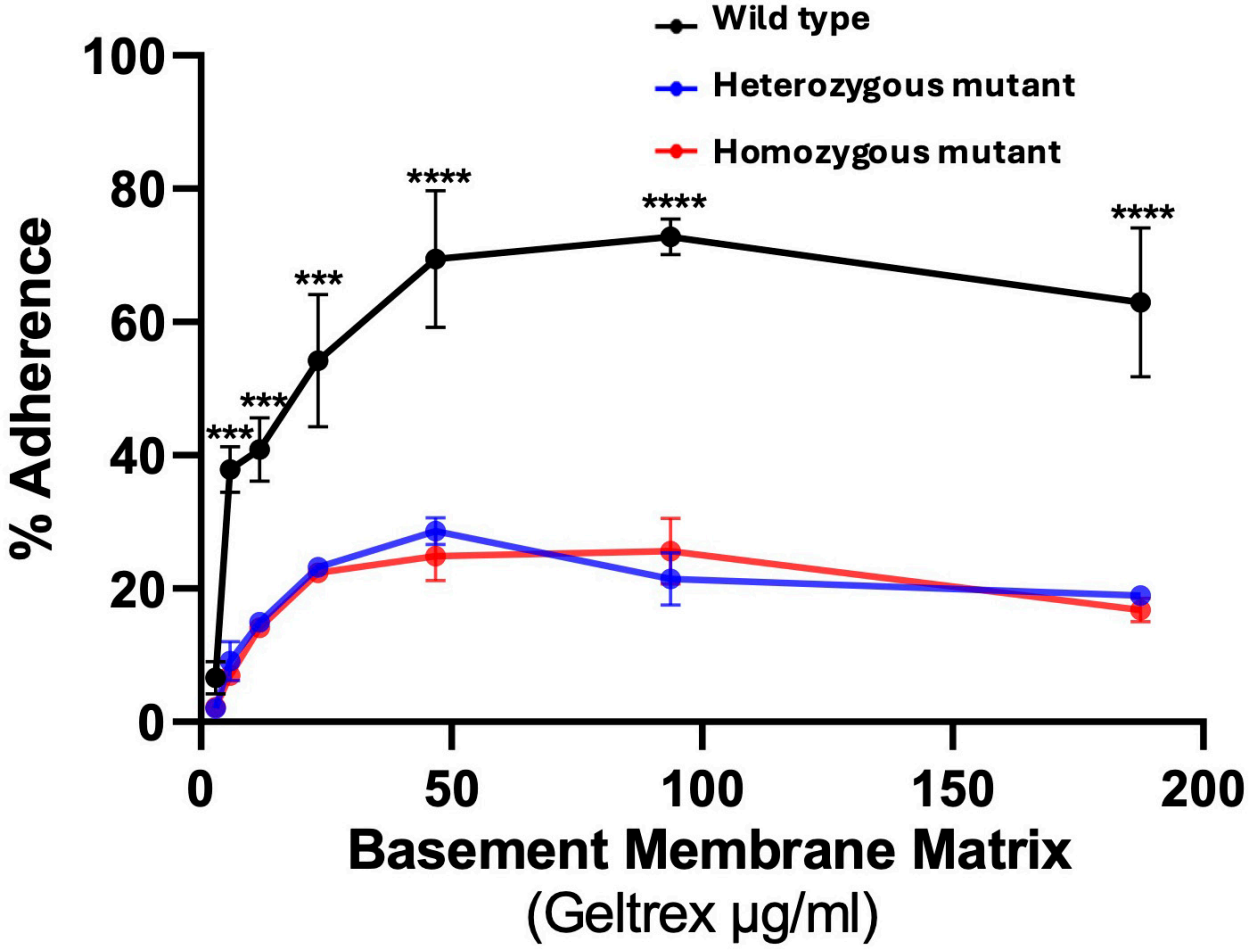
Isogenic *CTBP1* heterozygous and homozygous mutant cells adhere less than the parental wild type. NSCs were plated on black 96-well plates coated with varying concentrations of Geltrex, and adherence was assessed by the flipping assay. The percentage of adhered (flipped) cells was calculated based on un-flipped cells. Compared to the wild type, heterozygous and homozygous mutants showed significantly lower adherence on 5, 11, 23, 46, 93 and 187/μg/mL Geltrex-coated surfaces. No significant difference was found between the heterozygous and homozygous mutants. Three independent experiments were performed. Each experiment consisted of three replicates from each cell line. Data are presented as the mean ± standard error of the mean (SEM). *P* < 0.05 was considered significant, *n* = 3. ***p < 0.001, ****p < 0.0001.

Previously published data on iPSCs of the adhesion assay of patient-derived cells showed that mutant cells had lower adhesion compared to wild-type cells (Vijayalingam et al., 2020). To determine whether the isogenic wild-type had more adhesion than the *CTBP1* mutant NSCs, we conducted an *in vitro* “flipping” assay (Langhe et al., 2016). The percentage of adhered (flipped) cells was calculated based on the number of un-flipped cells. Compared to wild-type NSCs, *CTBP1* heterozygous and homozygous mutant NSCs showed significantly lower adherence for all tested Geltrex (basement membrane matrix) levels (5, 11, 23, 46, 93, and 187 μg/mL) as shown in Figure 3. Both the heterozygous and homozygous mutant NSCs exhibited similar levels of adherence; however, both mutants consistently showed a significantly lower percentage of adherence than the wild-type NSCs at all tested basement membrane matrix concentrations (Figure 3). Reduced adherence of mutant NSCs suggests dysregulation of adhesion molecules such as integrins and cadherins in mutant cells.

#### 3.3.2 Migration (wound healing/scratch assay)

A wound healing/scratch assay was conducted to determine whether the *CTBP1* mutation dysregulates NSC migration. Confluent cells in a 6-well plate were scratched using a pipette tip, and NSC migration in the scratch/wound area was measured by taking photographs at time points 0, 24, and 48 h.

**FIGURE 4:**
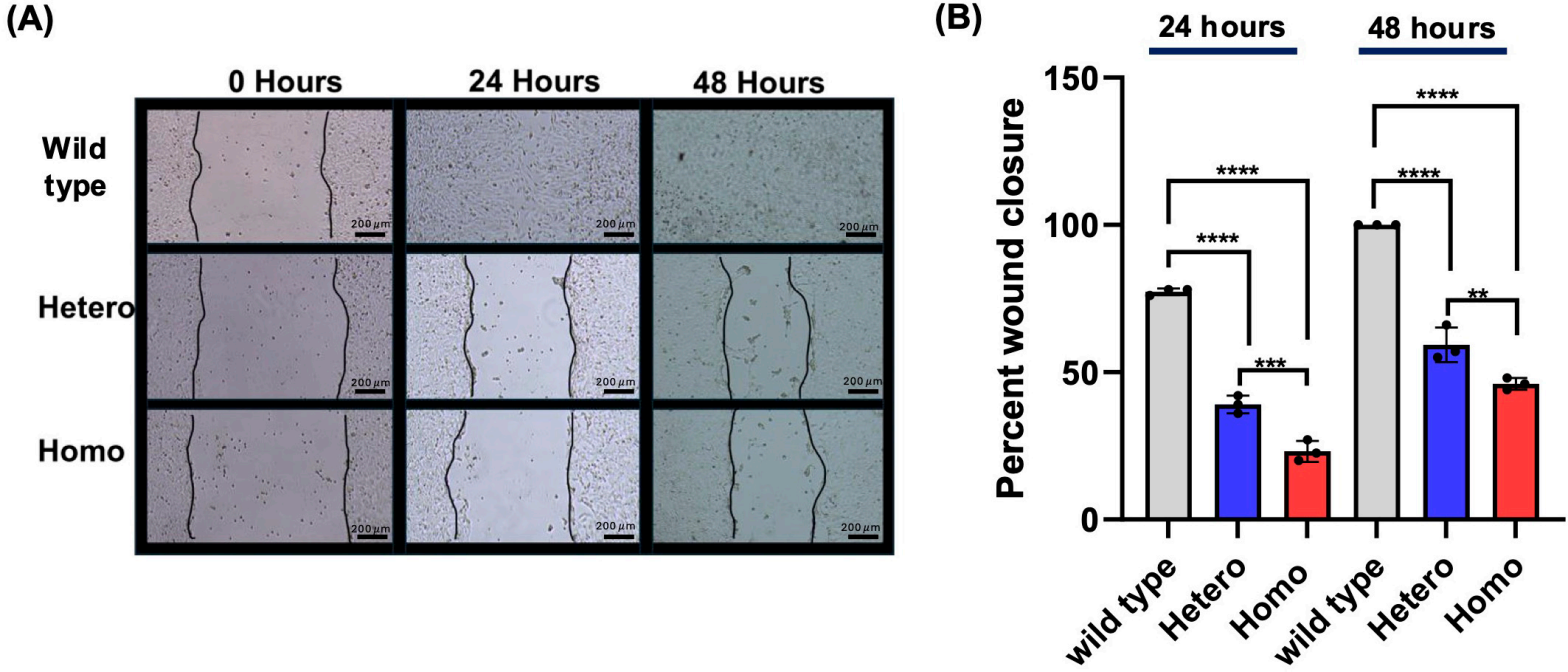
Cell migration and wound healing assays. Isogenic NSCs showed significantly less cell migration in the mutant cells compared to the wild-type cells. **(A)** Photograph shows migration of NSCs in mutant cells compared to wild-type cells at 0, 24, and 48 h. **(B)** The graph shows the percentage of wound closure at 24 and 48 h. Three independent experiments were performed, and each experiment consisted of duplicates from each cell line. *n* = 3, **p < 0.01, ***p < 0.001, ****p < 0.0001. Scale bar: 200 μm (4×).

Based on Figure 4, after 24 h, the wild-type wound had mostly closed; however, the gaps for the heterozygous and homozygous mutant NSC wounds were still apparent. A proliferation assay was conducted to ensure that cell growth was not a confounding factor for the migration of the cells (Supplementary Figure 7). We found that there were no significant differences between the cell proliferation of the three cell lines (Supplementary Figure 7). Therefore, the closure of the wound is not associated with cell proliferation.

Compared to isogenic wild-type NSCs, both heterozygous and homozygous mutant NSCs exhibited significantly decreased migration in the wound healing assay at both time points (Figure 4). The homozygous mutants exhibited significantly decreased migration in comparison to the heterozygous mutants. These results suggest that when both alleles harbor a *CTBP1* mutation, NSC migration is more severely affected.

#### 3.3.3 Migration and neurite formation in neurosphere assay

**FIGURE 5:**
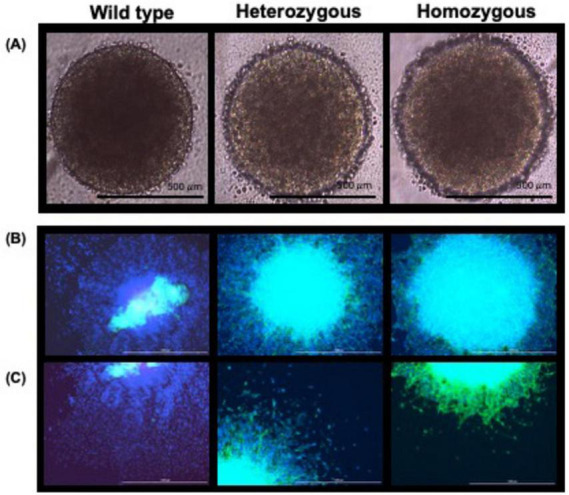
Neurosphere assay. **(A)** Neurospheres grown in StemPro NSC SFM media for 48 h are shown. Scale bar: 500 μm (4×). **(B)** Differentiated neurospheres fixed and stained with DAPI (blue) and MAP2 (green) on day 5 of differentiation. Scale bar: 1,000 μm (4×). **(C)** The edge of the neurosphere from panel B is shown for clarity. The high level of cell migration and spread was exhibited in the wild-type, compared to the heterozygous and homozygous neurospheres, respectively. A high level of neurite formation was observed in homozygous mutant neurons compared to the heterozygous mutant neurons, and very few neurites were formed in wild-type neurons. Scale bar: 1,000 μm (4×).

NSC neurosphere formation is an *in vitro* culture system used to study neurogenesis and neural development. This model allows the three-dimensional (3D) expansion of NSCs within a more physiologically relevant microenvironment (Galiakberova and Dashinimaev, 2020; Soares et al., 2021). The neurospheres can be differentiated into neurons, astrocytes, and oligodendrocytes, and the spreading and migration of neurospheres can be studied with all three of these cell types (Galiakberova and Dashinimaev, 2020; Soares et al., 2021). We qualitatively assessed cell migration and neurite formation of neurons with the neurospheres (Figure 5).

The fluorescent images (Figure 5) show the spread of the neurospheres that were differentiated from NSCs to neurons. As all neurospheres were formed with the same cell number and density, differences in neurosphere growth cannot be attributed to proliferation (Supplementary Figure 8). As seen in Supplementary Figure 8, for each experiment, the mutant diameter was normalized to the wild-type diameter. There were no statistically significant differences seen between the wild-type and mutant diameters for all three experiments. Additionally, as cells were grown in 96-well low adherence U-bottom plates (Biofloat, Sarstedt), the neurospheres were similar in size within each experiment.

The images show greater migration of the neurons from the center of the neurosphere in the wild-type neurons when compared to the mutant neurons. We performed immunocytochemistry of the neurospheres, where the nuclei were stained with DAPI (blue) and the neurites were stained with MAP2 (green) (Figure 5).

As shown in Figure 5, in wild-type neurospheres, a large number of cells migrated outwardly as can be seen by the DAPI staining and very few neurites were stained. This suggests that the neurites had not yet been extensively developed by wild-type neurospheres on day 5 of differentiation. On the other hand, more neurites and less cell migration were observed in heterozygous and homozygous mutant neurospheres as the neurons seem to be mostly clustered toward the center of the neurosphere with less observed migration when compared to the wild type (Figures 5B,C). These results indicate that the *CTBP1* mutation inhibits cell migration and leads to neurites being formed prematurely, as shown by MAP2 staining (Figures 5B,C) in the *CTBP1* mutant cells. Compared to the heterozygous mutants, the homozygous mutants did not exhibit apparent cell migration, but their neurites appeared more abundant.

#### 3.3.4 Neurite length

**FIGURE 6:**
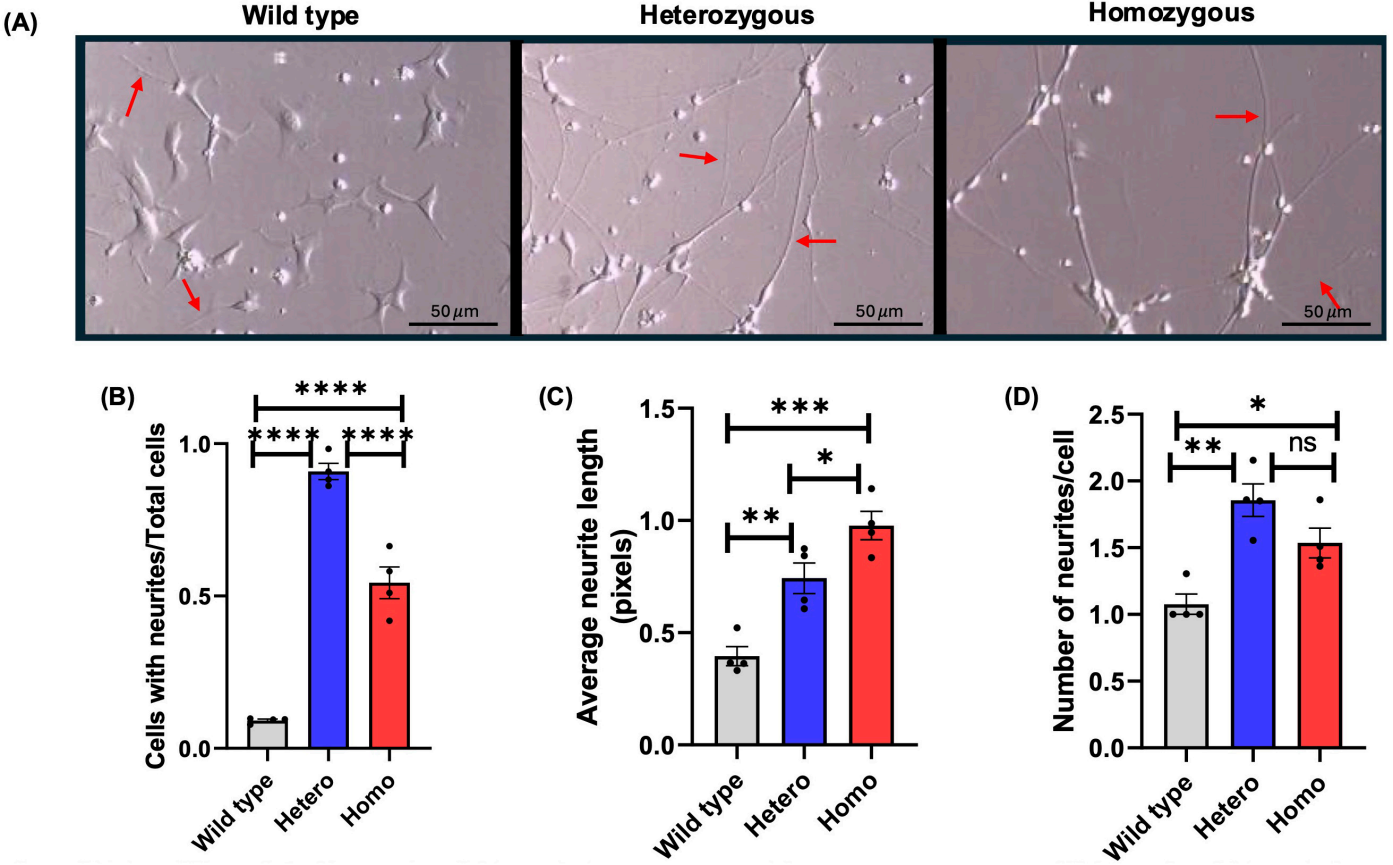
Neurite formation of 7-day differentiated isogenic wild-type, heterozygous, and homozygous neurons**. (A)** Isogenic wild-type, heterozygous mutant, and homozygous mutant neuron images taken on day 7 of differentiation. Long neurites are evident only in *CTBP1* mutant cells. The arrows indicate examples of neurites that were measured. **(B)** Cells with neurites are shown in comparison to the total number of cells. In both mutants, many cells had significantly more neurites compared to the wild type. The heterozygous mutants significantly exhibited more neurites compared to the homozygous and wild type. **(C)** Using ImageJ, neurite lengths and their averages were determined. Average neurite length was significantly higher in mutant cells compared to wild-type cells, and there was no significant difference between the mutants. **(D)** The number of neurites present in each cell containing neurites was calculated. Significantly more neurites were present in neurons in mutant cells compared to wild-type cells. There was no significant difference between the mutant cells. Four independent experiments were performed. In each experiment, for each cell line, images were acquired from three wells and analyzed, *n* = 4. *p < 0.05, **p < 0.01, ***p < 0.001, ****p < 0.001, ns, not significant. Scale bar: 50 μm (20×).

In the neurosphere experiments, we found that after 5 days of differentiation, neurites were formed in *CTBP1* mutant cells. To evaluate whether *CTBP1* mutant neurons formed neurites prematurely, we differentiated the isogenic wild-type and *CTBP1* mutant NSCs. On day 7 of neuron differentiation, we measured the number of cells with neurites, average length of neurites, and number of neurites per cell (Pemberton et al., 2018). Neurites were observed in all three cell lines, but heterozygous and homozygous mutant neurons had visibly longer neurites (Figure 6A).

Both *CTBP1* mutant neurons had a significantly higher ratio of cells with neurites per total cells when compared to wild-type neurons. However, between the mutants, heterozygous mutants had a significantly higher ratio of cells with neurites per total cells when compared to homozygous mutants (Figure 6B). Both mutant neurons had significantly longer average neurites than the wild type, with the homozygous mutants displaying greater neurite length than the heterozygous mutants (Figure 6C). There were also significantly more neurites per cell in both mutants compared to wild-type neurons, while there was no significant difference between the number of neurites per cell between the mutants (Figure 6D). These results suggest that the mutation increases early neurite formation with longer neurite lengths. Based on this experiment, we can conclude that *CTBP1* mutation causes dysregulation of neurite formation in both mutant neurons.

#### 3.3.5 Calcium transients

**FIGURE 7:**
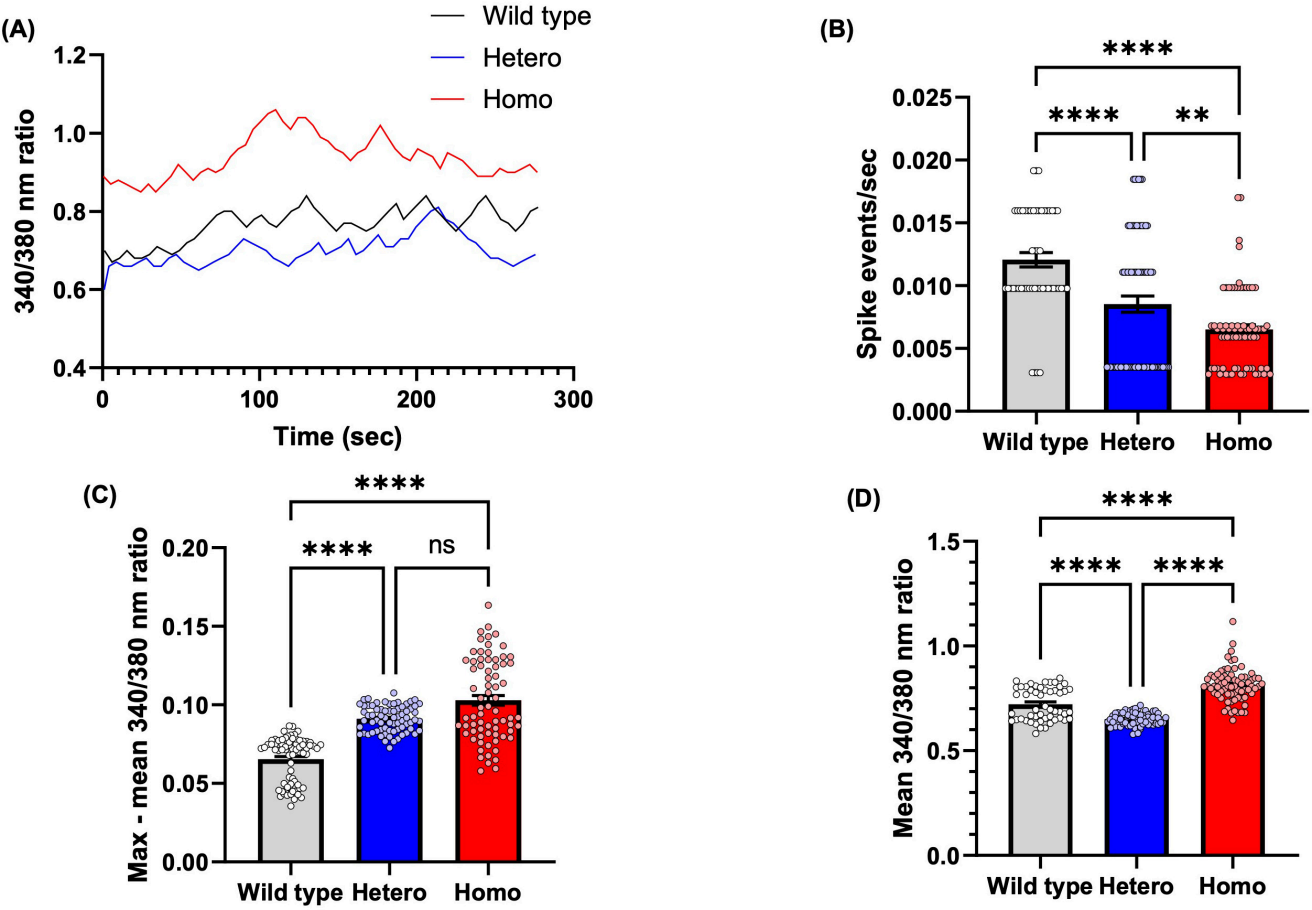
*CTBP1* mutation dysregulates NSC intracellular calcium. **(A)** Representative traces of spontaneous calcium spike events (calcium transients) measured as changes in Fura-2 ratiometric fluorescence intensity at 340/380 nm. **(B)** Spike events per second were measured to quantify spike frequency. Mutant cells had significantly lower spike events/sec than the wild-type cells. **(C)** The maximum amplitude of intracellular calcium, as determined by peak increase in 340/380 nm ratio compared to mean. Mutant cells had significantly higher max–mean 340/380 nm ratio than the wild-type cells. **(D)** The mean baseline intracellular calcium level, as determined by mean 340/380 nm ratio through the recording. The mean basal 340/380 nm ratio was significantly lower in heterozygous and significantly higher in homozygous compared to wild-type NSC. Only cells with at least one spike event were included in analysis (*N* = 52 cells from 3 experimental replicates for wild-type group, *n* = 72 cells from 2 to 3 experimental replicates for all other groups). **p < 0.01, ****p < 0.0001, ns, not significant.

In both isogenic NSC *CTBP1* mutants, migration and adhesion were affected (Figures 3–5), indicating that the mutation can affect key steps in neurodevelopment. NSC cellular functions are controlled by extracellular signals, many of which function by recruiting Ca^2+^, which acts as a secondary messenger. Ca^2+^ signaling can shape a wide range of cellular functions, including proliferation, migration, neurite outgrowth, and differentiation (Berridge et al., 2003). This highlights the importance of understanding NSC Ca^2+^ transients in elucidating early neurodevelopment. Many extrinsic and intrinsic factors play significant roles in the development of the central nervous system (CNS) through NSC membrane receptors, such as GPCRs and RTKs, that elicit intracellular Ca^2+^ transients in non-excitable cells (Diaz-Pina et al., 2024). To assess the effect of *CTBP1* mutation on Ca^2+^ transients, we performed fluorescent Ca^2+^ imaging experiments on wild-type and mutant NSCs (Figures 7A–D).

The 340/380 nm ratio reflects the cytoplasmic Ca^2+^ levels at baseline or during spiking activity, as shown in the raw traces in Figure 7A. Statistical analyses revealed that, compared to wild-type NSCs, CtBP1 mutant NSCs exhibited significant alterations in spiking frequency (Figure 7B), peak amplitudes (Figure 7C), and basal Ca^2+^ levels (Figure 7D). Specifically, wild-type NSCs showed a significantly smaller amplitude of spike events compared to both mutant NSC lines, and heterozygous NSCs had a significantly smaller amplitude compared to homozygous NSCs (Figure 7C).

Changes in Ca^2+^ spike patterns of NSCs during CNS development will determine the fate of the cells, especially radial glial cells that migrate along radial glial fibers to different zones of the brain during neurodevelopment (Diaz-Pina et al., 2024). Wild-type NSCs exhibited a significantly higher frequency of spike events compared to both heterozygous and homozygous NSCs. Additionally, heterozygous NSCs had a significantly higher frequency of spike events compared to homozygous NSCs. Both mutant NSCs had significantly lower spike events/second than wild-type NSCs (Figure 7B), which suggests that the *CTBP1* mutation deregulates Ca^2+^, thereby affecting cell migration, which is consistent with our findings from the scratch assay and the neurosphere migration assay.

Finally, basal Ca^2+^ levels also differed significantly among the cell lines. The mean baseline 340/380 nm ratio was significantly lower in heterozygous NSCs and significantly higher in homozygous NSCs compared to wild-type NSCs (Figure 7D) Together, these data reveal that CtBP1 mutant NSCs exhibit dysregulated basal Ca^2+^ levels and aberrant Ca^2+^ spike activity, characterized by less frequent but larger-amplitude spike events compared to wild-type NSCs.

## 4 Discussion

Transcriptional corepressor *CTBP1* in the heterozygous condition with a specific R342W mutation causes prominent neurodevelopmental manifestations, including hypotonia, ataxia, and developmental delays in HADDTS patients (Acosta-Baena et al., 2022; Beck et al., 2016; Beck et al., 2019; Vijayalingam et al., 2020). The neurodevelopmental phenotypes observed in these patients due to the *CTBP1* p.R342W allele provide genetic evidence that *CTBP1* is critical for normal human neurodevelopment.

Due to CtBP1’s complex function as a transcriptional corepressor (Chinnadurai, 2002) that regulates the expression of numerous target genes, the phenotypic differences observed in the mutant lines are likely driven by broad, genome-wide transcriptomic alterations rather than a suppression of a single gene expression. In this study, to examine the effects of CtBP1 mutations on NSC and neuronal function, we utilized a heterozygous and homozygous isogenic cell line.

STR profiling confirmed > 90% concordance between the parental and gene-edited lines, consistent with ATCC authentication standards indicating shared origin (Capes-Davis et al., 2013; Almeida et al., 2016). The single allelic difference observed at one of the 15 tested loci is likely attributable to parental mosaicism or to spontaneous allelic drift caused by polymerase slippage that can occur during cell culture or clonal expansion (Hameed et al., 2024; Supplementary Figure 1). According to the International Cell Line Authentication Committee (ICLAC), ≥ 80% concordance across 13 STR loci is sufficient to confirm relatedness, and our analysis included 15 loci, further strengthening the validation of cell line identity (Almeida et al., 2016).

In this study, using isogenic cell lines, we compared some neurodevelopmental processes and the neurite morphology of mutant to control cells. Similar to our previous observations (Vijayalingam et al., 2020), we found morphological differences in heterozygous mutant neurites characterized by a punctated appearance as stained by beta-3 tubulin (Figure 1).

Although the major role of CtBP1 is transcriptional corepression, CtBP1 is also known to control synaptogenesis and act as a regulator of membrane fission (Ivanova et al., 2020). In neurons, CtBP1 occurs in two major defined pools, in the nucleus and the presynaptic compartment. The redistribution is controlled by intracellular NAD/NADH levels. It has been shown that based on the cell activity (NAD/NADH level), CtBP1 translocates between the synapses and cell body (Ivanova et al., 2020). At presynaptic structures, CtBP1 interacts with two large scaffolding proteins, bassoon and piccolo (Ivanova et al., 2015). These proteins organize presynaptic terminals by anchoring synaptic vesicles and coordinating the release of neurotransmitters (Ivanova et al., 2015). Apart from CtBP1 traveling to the presynaptic terminal (anterograde translocation) and being retained by the scaffolding protein, it can also be transported back to the nucleus by axonal transport or facilitated diffusion (retrograde translocation) (Ivanova et al., 2015).

While the role of synaptic CtBP1 in neurons is unclear, we predict that a wt/mt CtBP1 dimer may interact with the transporting protein or diffusion mechanism and affect its translocation. As beta-3 tubulin is involved in the transportation of proteins from the cytoplasm to the pre-synaptic terminal, the beta-3 tubulin antibody can stain for the stalled presynaptic transport complexes which may be visible as puncta in the heterozygous neurons (Radwitz et al., 2022; Figure 1). As homozygous mutant cells only have a mt/mt CtBP1 dimer with no wild-type monomer being present, the formation of the complex with transport mechanisms is either affected or not formed (Figure 1). This could result in an absence in the puncta on the neurites of the homozygous mutants.

As isogenic cell lines have the same genetic background except for the mutation in CtBP1, we can compare the common genes expressed in heterozygous and homozygous mutant cells to find the key genes affected by the *CTBP1* mutation. Our hypothesis is that the downregulated genes will be more suppressed in the homozygous than the heterozygous mutants. The fold-down regulation was calculated by finding the ratio of the logFC value of the homozygous mutant divided by the heterozygous mutant’s expression levels. We found 26 genes that were more highly suppressed in the homozygous compared to the heterozygous mutants; among the 26, 10 were TFs, and one was a transcription repressor (Table 1). We hypothesize that *CTBP1* mutation affects the expression of these critical TFs, thereby impacting neurodevelopment.

Based on the Allen Brain Cell atlas, although *CTBP*s are expressed throughout the brain, the highest expression of *CTBP1* was localized in the upper rhombic lip (URL), which gives rise to cerebellar cells, including cerebellar granule cells (CGCs) (Lee and Ezekiel, 2025). In both the heterozygous and homozygous *CTBP1* mutant transcriptomes, we found that TFs critical for CGC growth and development are downregulated. Likewise, as seen in Table 1, some of the TFs with a > 1.5-fold logFC downregulation in homozygous compared to heterozygous mutants are involved in URL development. *OLIG3* is involved in the development of the earliest stage of rhombic lip development (Takebayashi et al., 2000). An *OLIG3* mutation in mice has been shown to cause cerebellar hypoplasia and the loss of early-born neurons (Lowenstein et al., 2021). *BARHL1* is involved in the development of CGCs (Li et al., 2004). Based on the higher fold downregulation of these two genes in homozygous mutant neurons compared to heterozygous mutant neurons (Table 1), we suggest that the defects observed in HADDTS patients may be due to CGC dysfunction.

Apart from the TFs involved in cerebellar development, as shown in Table 1, we identified other TFs that are also involved in neurodevelopment and brain function. Some examples of TFs that are highly downregulated in homozygous compared to heterozygous mutant neurons are GATA binding protein 3 (*GATA3*), *HEY2* (Hes-related Family BHLH Transcription factor with YRPW motif), *ISL1, NKX2-2, SP9*, and *ARX*. Mice homozygous for a targeted disruption of *GATA3* died between embryonic days 11 and 12 with severe deformities of the brain, spinal cord development, and fetal liver (Pandolfi et al., 1995). In mouse retinal neurocytes, silencing of *GATA3* expression promoted neuronal differentiation and neurite outgrowth (Chen et al., 2022). Our data suggests that neurites are formed earlier in *CTBP1* mutants than in the wild type, possibly due to the low level of *GATA3* in mutant cells (Table 1). Dysregulation of *HEY2* is implicated in cardiac and neurodevelopmental defects. Individuals with chromosomal deletion of *HEY2* exhibit cardiac defects and cognitive impairment, and duplication of *HEY2* leads to congenital heart defects and neurodevelopmental delays (Jordan et al., 2015). In mice, misexpression of *HEY2* in the brain at 13.5 days led to the dysregulated maintenance of neural precursors and an increase in late-born neurons. However, *HEY2* expression on day 15 led to the inhibition of neurogenesis and promotion of glycogenesis (Sakamoto et al., 2003). Insulin gene enhancer protein (*ISL1*) is a LIM-homeodomain TF (Zhang et al., 2018). *ISL1* was also downregulated in human iPSC-derived neurons from *CTBP1*-mutated patients (Vijayalingam et al., 2020). Hypomorphic mice with reduced *ISL1* expression exhibit dysregulated sympathetic neuron differentiation and decreased expression of genes involved in axonal growth and neurotransmission expression (Filova et al., 2022). Inner ear development is affected in *ISL1* knockout mice, with a reduced cochlear nucleus and defective migration of spiral ganglion neurons (Filova et al., 2022).

Exposing the dorsoventral axis of the neural tube to different gradients of Shh leads to the activation of transcriptional regulatory proteins that act as an activator or repressor, forming distinct progenitor cell populations (Briscoe, 2009). Using knockout mice that are double mutants for *NKX2-2* and *NKX2-9*, it is observed that both TFs play unique and redundant functions. *NKX2-2* is involved in the development of distinct neuronal populations in the hindbrain and ventral spinal cord, as well as severe axonal pathfinding defects in commissural neurons (Holz et al., 2010). The majority of the double mutants also exhibited abnormal migratory defects (Holz et al., 2010). *SP9* regulates median ganglionic eminence-derived interneuron development and the expression of *LHX6*, which regulates the tangential migration of median ganglionic eminence-derived interneurons (Liu et al., 2019). In humans, mutations in *ARX* lead to intellectual disability (Colombo et al., 2004). *ARX* controls cortical interneuron differentiation and migration by regulating genes involved in proliferation, cell cycle, and migration (Lim et al., 2024).

Neurodevelopmental processes involve several key steps which include adhesion, cell migration, and differentiation (Jiang and Nardelli, 2016). Migration and adhesion are interrelated, and there are several genes that affect both processes. In our experiment, we found that adhesion, as measured by the flipping assay, was significantly affected in both mutants compared to the wild type. In migration, as measured by the wound healing assay, although both mutants significantly migrated less compared to the wild type, homozygous mutants exhibited more severe migration defects than heterozygous mutants (Figures 3, 4). In our neurosphere assay, we found that cell migration was inhibited in both mutants compared to the wild type with homozygous mutants being more profoundly affected (Figure 5). CtBPs have been shown to play a critical role in the development and cell survival by suppressing pro-apoptotic genes, regulating cell movement and adhesion molecules, and allowing cells to undergo EMT (Grooteclaes et al., 2003). EMT is involved in cell migration during embryonic development, neural tube formation, and metastasis of tumors (Francou and Anderson, 2020). During EMT, cells undergo cytoskeletal reorganization and acquire migratory phenotypes (Francou and Anderson, 2020). CtBP1 is shown to be a critical player in the EMT process by modulating cell adhesion genes (Zhang et al., 2013). Cell adhesion and migration depend on the expression of integrins that interact with extracellular matrix which in turn initiate cellular responses through signaling pathways (Morante-Redolat and Porlan, 2019). Forkheadbox 1 (*FOXF1*) is a TF that is downregulated in both mutants (Supplementary Table 1) and mediates integrin beta-3 expression, that plays a vital role in interactions between the extracellular matrix and other cells (Malin et al., 2007).

We found that both heterozygous and homozygous mutant NSCs exhibited significantly less adherence than the wild type (Figure 3). Migration was also significantly inhibited in both *CTBP1* NSC mutants compared to the wild-type control. The Shh pathway plays a critical role in cell adhesion and migration. *SHH* expression is 2.3-fold downregulated in *CTBP1* homozygous mutants compared to heterozygous mutants (Supplementary Table 2). Both in *CTBP1* heterozygous and homozygous mutants, *NKX2-2, SULF1*, and *ARHGAP36*, which are involved in the Shh pathway, are downregulated (Supplementary Table 1). As a morphogen, Shh plays a pleiotropic role during embryogenesis, neuronal patterning, and axon guidance (Douceau et al., 2023; Ishibashi and McMahon, 2002; Peng et al., 2018).

During development, the secreted gradient of Shh, which is interpreted by downstream TFs, leads to the specification of different progenitor cells during neurodevelopment (Ribes et al., 2010). Shh-induced signaling on CGC proliferation is critical for the proper fissure formation of the cerebellum (Corrales et al., 2006). *NKX2-2* acts in response to Shh and is required for the ventral floor plate and P3 progenitors in the ventral neural plate (Lek et al., 2010). *SULF1* encodes a cell surface sulfatase that removes specific 6-O-sulfate groups from heparan sulfate (HS) proteoglycans, which leads to an HS-dependent signaling pathway (Kalus et al., 2009). *SULF1* modulates the Shh signaling pathway, and a reduction in *SULF1* expression has been shown to inhibit Shh signaling (Kalus et al., 2009). Rho GAP family member *ARHGAP36* also regulates Shh activity by inhibiting PKA activity and is involved in lateral motor column neurons (Nam et al., 2019). Downregulation of Shh, a critical morphogen, can affect several neurodevelopmental processes. Therefore, transcriptome analysis supports the adhesion and migration defects observed in the mutant NSCs.

We observed that neurites are formed much earlier in differentiated neurons than in the wild-type control (Figures 5, 6). Compared to the total number of cells, *CTBP1* heterozygous mutant cells had a significantly greater number of cells with neurites than homozygous mutant and wild-type neurons (Figure 6B). The average neurite length was significantly greater in both mutants compared to the wild type, homozygous mutants displaying greater neurite length than the heterozygous mutants (Figure 6C). We also found that both mutants exhibited significantly higher numbers of neurites per cell when compared to wild-type cells (Figure 6D). These results also correlate with our transcriptome analysis. TFs *GATA3, NKX2-2*, and *ISL1* regulate neurite formation and axonal growth (Bhardwaj et al., 2024; Chen et al., 2022; Holz et al., 2010). These results suggest that the *CTBP1* mutation suppresses genes that regulate neurite formation, supporting the results we have seen in dysregulated neurites in mutant neurons (Figures 5, 6). Transcription factor AP-2 gamma (*TFAP2C)* is involved in neural crest induction *(Li and Cornell, 2007). TFAP2C* (Supplementary Table 1) is downregulated in both *CTBP1* heterozygous and homozygous mutant neurons and plays a critical role in directing the outgrowth and branching of neurites (Zhao et al., 2023). *TFAP2C* targets miR-132-5p, which has been known to inhibit extensive neurite outgrowth and to regulate neurite extension (Zhao et al., 2023). Therefore, the downregulation of *TFAP2C* may be involved in the neurite outgrowth seen in mutant neurons. *CTBP1* mutation affects TFs (Table 1), which in turn dysregulate target gene expression, thereby disrupting multiple neurodevelopmental processes.

During neurodevelopment, Ca^2+^ fluctuations play a critical role in cell migration, adhesion, and neurite formation (Toth et al., 2016). Ca^2+^ spikes, transients, or oscillations are critical for understanding intracellular Ca^2+^ signaling. Rhythmic Ca^2+^ oscillations in developing cells can direct specific steps of neurodevelopment (Toth et al., 2016). In general, oscillations in Ca^2+^ signals can be more effective in driving effector responses than sustained Ca^2+^ signals due to the high signal-to-noise discrimination (Alswied and Parekh, 2015). Ca^2+^ entry into NSCs is crucial for cell cycle regulation, and depending on the activated pathway, NSCs will either self-renew or differentiate into specific neural lineages (Forostyak et al., 2013; Fukuoka et al., 2024).

Our Ca^2+^ imaging experiments showed that wild-type NSCs exhibited a significantly higher frequency of spike events than the heterozygous and homozygous mutants (Figure 7B). The mean intracellular Ca^2+^ influx level, measured as the change in the 340/380 nm ratio, in homozygous mutant NSCs exhibited a greater mean 340/380 nm ratio than other NSC counterparts, indicating a high amplitude of intracellular Ca^2+^ transients (Figure 7C). The dysregulated Ca^2+^ levels can also affect cell adhesion and migration.

The relatively higher max – mean 340/380 nm ratios in the heterozygous and homozygous mutant cells compared to the wild-type cells may be a compensatory result. A high level of Ca^2+^ and low spike event can inhibit cell migration, supported by our results which show that both heterozygous and homozygous mutant cells are significantly inhibited compared to wild-type NSCs (Toth et al., 2016). Transient elevations in Ca^2+^ levels are involved in neuronal migration, as the amplitude and frequency of Ca^2+^ fluctuations are positively correlated with CGC movement in cerebellar microexplant cultures (Komuro and Rakic, 1996). Cells exhibit the greatest migration activity during each Ca^2+^ elevation, indicating that the frequency or occurrence of Ca^2+^ transients, rather than their amplitude, is more closely associated with temporal cell movement (Komuro and Rakic, 1996; Komuro and Kumada, 2005). This interpretation is consistent with our findings that wild-type NSCs, which display more frequent but smaller-amplitude Ca^2+^ spikes, migrate more efficiently than mutant NSCs that exhibit less frequent but larger-amplitude events. The altered Ca^2+^ signaling pattern in mutant cells likely reflects perturbations in Ca^2+^ handling, such as dysregulation of ion channels, intracellular stores, or buffering mechanisms, which in turn contribute to impaired cell migration and neural developmental processes (Komuro and Kumada, 2005).

Supporting this notion, transcriptomic analysis revealed that several ion channel-related genes involved in Ca^2+^ regulation were dysregulated in mutant cells compared to wild-type cells, and even more drastically dysregulated in homozygous compared to heterozygous mutant neurons (Supplementary Tables 1, 2). Among these, *BEST3* encodes Bestrophin 3, a protein that mediates cGMP-dependent Ca^2+^ activated chloride conductance in vascular smooth muscles. Notably, a mutation in this gene has been linked to Vitelliform Macular Dystrophy 2–Like 3 (Matchkov et al., 2008). The BEST family member, *BEST1*, which is upregulated in injured sensory neurons, is involved in the nerve regeneration process (Boudes et al., 2009). Based on the downregulation of *BEST3*, we suggest that this gene may play a role in neurodevelopmental processes. In addition, *BEST3* encodes a pentameric, Ca^2+^-activated chloride channel (CaCC) that modulates membrane potential and influences Ca^2+^ signaling in nervous tissue as well as in other excitable or non-excitable cells (Owji et al., 2021; Golubinskaya et al., 2019; Pifferi et al., 2009; O’Driscoll et al., 2008). Disrupted expression likely impairs Ca^2+^-dependent chloride flux, thereby compromising the cell’s ability to restore the baseline membrane potential and maintain Ca^2+^ homeostasis and dynamics, as shown in Figure 7. Figures 7A,D further show that the basal 340/380 nm ratio is consistently higher in homozygous mutants than in heterozygous or wild-type cells. Consistent with these findings, we observed that *GABRA3*, which encodes the alpha-3 subunit of the GABA-A receptor (a ligand-gated chloride channel), was downregulated in homozygous mutants while upregulated in heterozygous mutants (Supplementary Table 1). As GABA-A receptor activation is known to reduce neuronal membrane excitability and inhibit calcium influx (Heidelberger and Matthews, 1991; Marlin and Carter, 2014), the downregulation of *GABRA3* in homozygous cells and its upregulation in heterozygous cells may underlie the elevated and reduced basal Ca^2+^ levels observed in Figures 7A,D.

In addition, defects in voltage-gated Ca^2+^ channels can lead to dysregulation of intracellular Ca^2+^ concentrations, thereby affecting neuronal function (Simms and Zamponi, 2012). In line with this, we found that *CACNG6*, which encodes an auxiliary subunit that regulates the inactivation state of voltage-gated Ca^2+^ channels (Arikkath and Campbell, 2003; Dolphin, 2016; Guan et al., 2016), is dysregulated in both homozygous and heterozygous NSCs. Altered *CACNG6* expression can directly affect Ca^2+^ channel conductance, resulting in aberrant Ca^2+^ spiking activity as observed in Figures 7A–C. Interestingly, dysregulation of genes encoding voltage-gated Ca^2+^ channels, such as *CACNA2D1*, was also observed in human iPSC-derived neuronal cells from *CTBP1*-mutated patients (Vijayalingam et al., 2020), underscoring CtBP1’s critical role in regulating gene networks that maintain neuronal calcium homeostasis and channel activity.

Adhesion defects in mutant cells also correlate with dysregulated Ca^2+^ signaling. Altered Ca^2+^ dynamics in mutant NSCs, including changes in spike frequency and amplitude, can lead to focal adhesion disassembly and reduce adhesion (Giannone et al., 2004). Elevated cytosolic Ca^2+^ can trigger diffusion of integrins out of the focal adhesions and activate calpain to disassemble adhesion complexes, as observed in adherent fibroblasts (Tsai et al., 2015). During migration, Ca^2+^ influx at the back end of the migrating cells helps detach rear ends and maintain a front-low, back-high Ca^2+^ gradient, which is critical for coordinated migration and adhesion (Giannone et al., 2004). This gradient is established in part by the polarized distribution of plasma membrane Ca^2+^ ATPase, which pumps more cytosolic Ca^2+^ out of the cell in the front than in the back (Strehler et al., 2007). Together, these mechanisms provide a mechanistic link between altered Ca^2+^ dynamics and impaired adhesion in *CTBP1* mutant NSCs.

Different types of cells respond to distinct frequency ranges of Ca^2+^ oscillations, highlighting the importance of temporal coding in intracellular Ca^2+^ signaling (Smedler and Uhlen, 2014). Moreover, Ca^2+^ oscillation waves are key regulators of gene expression, and precise regulation of Ca^2+^ signaling is critical for proper development and maintenance of cellular functions (Hannanta-Anan and Chow, 2016; Smedler and Uhlen, 2014). Our Ca^2+^ imaging results indicate that pathogenic *CTBP1* mutations dysregulate both the frequency and amplitudes of transients as well as basal levels, suggesting that proper neurodevelopment and function could be affected in HADDTS. Together these findings support a model in which *CTBP1* mutations lead to dysregulated Ca^2+^ signaling, resulting in impaired adhesion, altered migration, and early neurodevelopmental deficits.

**FIGURE 8:**
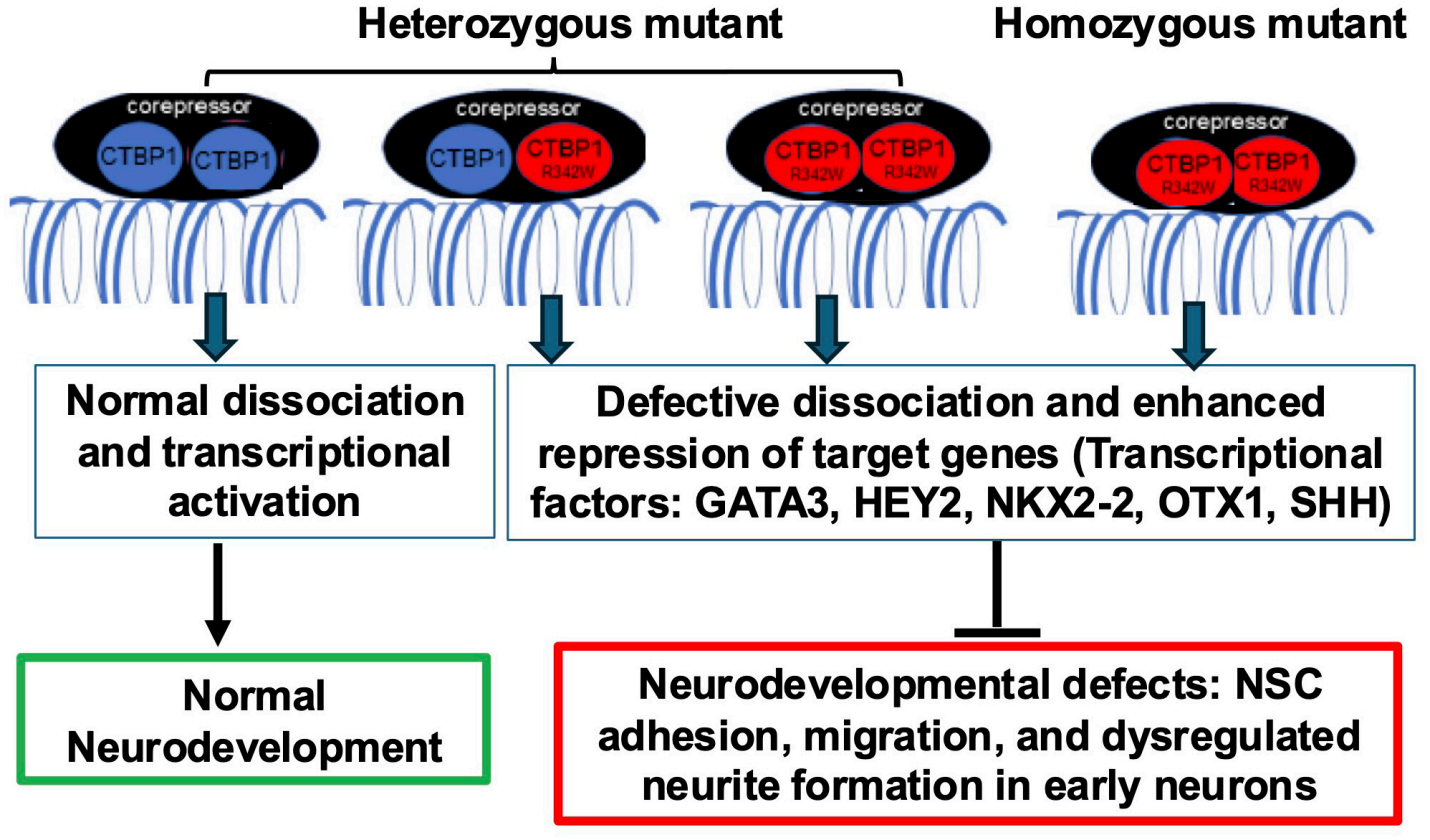
Model for repression of key transcription activators by the *CTBP1* missense mutation. In heterozygous mutant cells, mutant (mt)/wild-type (wt) and mt/mt CtBP1 dimerization cause defective dissociation and reduced expression of transcriptional activators vs. wt/wt dimer. In homozygous cells, only defective mt/mt dimers form, leading to greater suppression of transcriptional activators and more severe developmental defects compared to heterozygous mutants.

To understand how dysregulation of neurites, cell migration, and Ca^2+^ homeostasis are affected by the pathogenic mutation in heterozygous and homozygous conditions, we propose a working model (Figure 8). We hypothesize that mutant CtBP1 monomer(s) can form defective homodimeric or oligomeric complexes that can lead to sustained repression of neuronal target genes. This may occur either directly through impaired dissociation of the repressor complex from target gene promoters or indirectly through reduced expression of TFs (Vijayalingam et al., 2020). As shown in Figure 8, heterozygous mutant cells contain both normal and mutant CtBP1 proteins. The resulting repressor complexes, which include mutant monomers, may lead to delayed dissociation from chromatin and excessive repression of TFs. However, since approximately one-third of CtBP1 dimers in heterozygous cells are wt/wt, some repressor complexes retain normal function. In contrast, homozygous mutant cells form only mutant dimers, eliminating this balance and causing stronger suppression of TFs and their downstream target genes (Supplementary Table 1). Within the commonly expressed genes between mutants, homozygous mutants exhibited greater downregulation compared to the heterozygous mutants, which aligns with our proposed model.

Based on our *in vitro* assays, when both alleles are mutated, the phenotype is more severely affected as seen in NSC migration (Figure 4), the neurosphere assay (Figure 5), average neurite length (Figure 6), and calcium transient data (Figures 7B,C). The biological function of the candidate genes affected by the *CTBP1* mutation found in Table 1 can be explored in future studies. Although adhesion (Figure 3) and number of neurites per cell (Figure 6C) data did not show significant differences between the mutants, this may be due to the complex biological outcomes associated with the possible heterodimerization of CtBP1 and CtBP2 that contain mutant monomers.

Several studies now also propose that cerebellar hypoplasia and motoric abilities in HADDTS patients progressively worsen as patients age (Zhang M. et al., 2024; Zhang Q. et al., 2024). However, due to neuronal plasticity, the neurodevelopmental defects cellularly observed in patients may manifest later in life, peaking in young adulthood (Oberman and Pascual-Leone, 2013). Dysfunctional cerebellar circuits may also become more prone to miswiring as they are more vulnerable to metabolic stress as patients age (Verpeut and Oostland, 2024). Therefore, neurodevelopmental defects can manifest and progressively worsen later in life.

We hypothesize that the TFs affected by the *CTBP1* mutation cause several aspects of the neurodevelopmental processes, such as migration, adhesion, dysregulated neurite formation, and irregular Ca^2+^ homeostasis. Expression of one or a combination of these TFs (Table 1) could rescue one of these phenotypes. Based on the Allen Brain Cell atlas data, high expression of *CTBP1* in the URL gives rise to CGCs, as supported by rodent brain studies (Hamada et al., 2023; Lee and Ezekiel, 2025). Future studies will focus on differentiating isogenic iPSCs into CGCs or establishing a cerebellar organoid model to elucidate the specific role of *CTBP1*, thereby deepening our understanding of HADDTS. These efforts will be complemented by validation of the observed phenotypes across additional, independently derived isogenic *CTBP1* mutant and patient-derived cell lines.

## Data Availability

The datasets presented in this study can be found in online repositories. This data can be found here: https://www.ncbi.nlm.nih.gov/geo/query/acc.cgi?acc=GSE306099.

## Footnotes

1
http://rsb.info.nih.gov/ij/
