# Supplementary material for: Isogenic iPSC-derived CTBP1 mutant neuronal cells exhibit neurodevelopmental defects
Source: Front Neurosci. 2025 Dec 12;19:1695464. doi: 10.3389/fnins.2025.1695464 (PMC12741082; doi:10.3389/fnins.2025.1695464)
Supplement: Supplementary file 1 [file Data_Sheet_1.zip › High Resolution Supplementary/Supplementary Table_2 homo vs hetero.docx]

|  | **Gene name** | **logFC heterozygous mutant (Hetero)** | **adj. P. Value**  **Hetero** | **logFC homozygous mutant (Homo)** | **adj. P. Value Homo** | **logFC homo/hetero** |
| --- | --- | --- | --- | --- | --- | --- |
| 1 | **GATA3** | -2.14 | 7.90E-09 | -9.77 | 5.38E-06 | 4.565420561 |
| 2 | MRAP2 | -2.14 | 1.16E-02 | -6.39 | 1.68E-04 | 2.985981308 |
| 3 | AL672277.1 | -2.54 | 6.20E-04 | -7.2 | 2.61E-05 | 2.834645669 |
| 4 | HS3ST2 | -2.19 | 3.43E-02 | -5.71 | 5.35E-03 | 2.607305936 |
| 5 | **HEY2** | -3.37 | 1.89E-05 | -8.24 | 3.84E-03 | 2.445103858 |
| 6 | HAS2 | -3.1 | 8.07E-03 | -7.35 | 3.20E-02 | 2.370967742 |
| 7 | **BEST3** | -3.4 | 3.14E-04 | -7.85 | 1.18E-04 | 2.308823529 |
| 8 | **SHH** | -2.82 | 3.62E-07 | -6.39 | 2.80E-06 | 2.265957447 |
| 9 | **OTX1** | -2.3 | 9.39E-04 | -5.13 | 2.54E-04 | 2.230434783 |
| 10 | MT1X | -2.26 | 3.81E-03 | -4.7 | 8.32E-04 | 2.079646018 |
| 11 | RTL8A | 2.44 | 3.06E-07 | 4.67 | 7.08E-11 | 1.913934426 |
| 12 | **ISL1** | -4.32 | 1.12E-05 | -8.09 | 1.20E-04 | 1.872685185 |
| 13 | **NKX2-2** | -5.47 | 7.03E-09 | -10.1 | 1.78E-07 | 1.846435101 |
| 14 | ***INSM2*** | -2.19 | 5.89E-03 | -3.97 | 2.58E-03 | 1.812785388 |
| 15 | **SP9** | -2.16 | 5.61E-03 | -3.88 | 1.37E-03 | 1.796296296 |
| 16 | LINC01210 | -4.2 | 1.15E-05 | -7.35 | 1.20E-04 | 1.75 |
| 17 | **BARHL1** | -2.7 | 4.61E-02 | -4.66 | 2.69E-02 | 1.725925926 |
| 18 | HLA-B | 2.37 | 1.96E-07 | 4.06 | 1.56E-10 | 1.713080169 |
| 19 | MT1F | -2.5 | 4.03E-04 | -4.25 | 2.91E-04 | 1.7 |
| 20 | **ARX** | -2.68 | 2.89E-03 | -4.39 | 2.07E-03 | 1.638059701 |
| 21 | SLC18A2 | -2.18 | 1.85E-02 | -3.43 | 9.56E-03 | 1.573394495 |
| 22 | AC010478.1 | -3.57 | 6.00E-04 | -5.61 | 1.33E-03 | 1.571428571 |
| 23 | EIF1AY | -4.17 | 3.08E-04 | -6.48 | 6.15E-05 | 1.553956835 |
| 24 | **OLIG3** | -2.55 | 8.13E-03 | -3.93 | 9.92E-03 | 1.541176471 |
| 25 | BCAN | -2.24 | 1.79E-03 | -3.44 | 1.33E-03 | 1.535714286 |
| 26 | PALMD | -3.3 | 8.55E-08 | -5.02 | 1.40E-06 | 1.521212121 |

**Supplementary Table 2. Severely suppressed genes in homozygous mutants compared to heterozygous mutants.** Among the 26 genes listed with logFC homo/hetero greater than 1.5, 10 were transcriptional factors (bold), and one was a transcriptional repressor (*INSM2*, bold and italicized). There were no transcriptional factors with >1.5 logFC ratio found in upregulated genes, except the retrotransposon Gag-like 8A (RTL8A) which is derived from retrovirus.
