# Supplementary figures and images for: Isogenic iPSC-derived CTBP1 mutant neuronal cells exhibit neurodevelopmental defects
Source: Front Neurosci. 2025 Dec 12;19:1695464. doi: 10.3389/fnins.2025.1695464 (PMC12741082; doi:10.3389/fnins.2025.1695464)

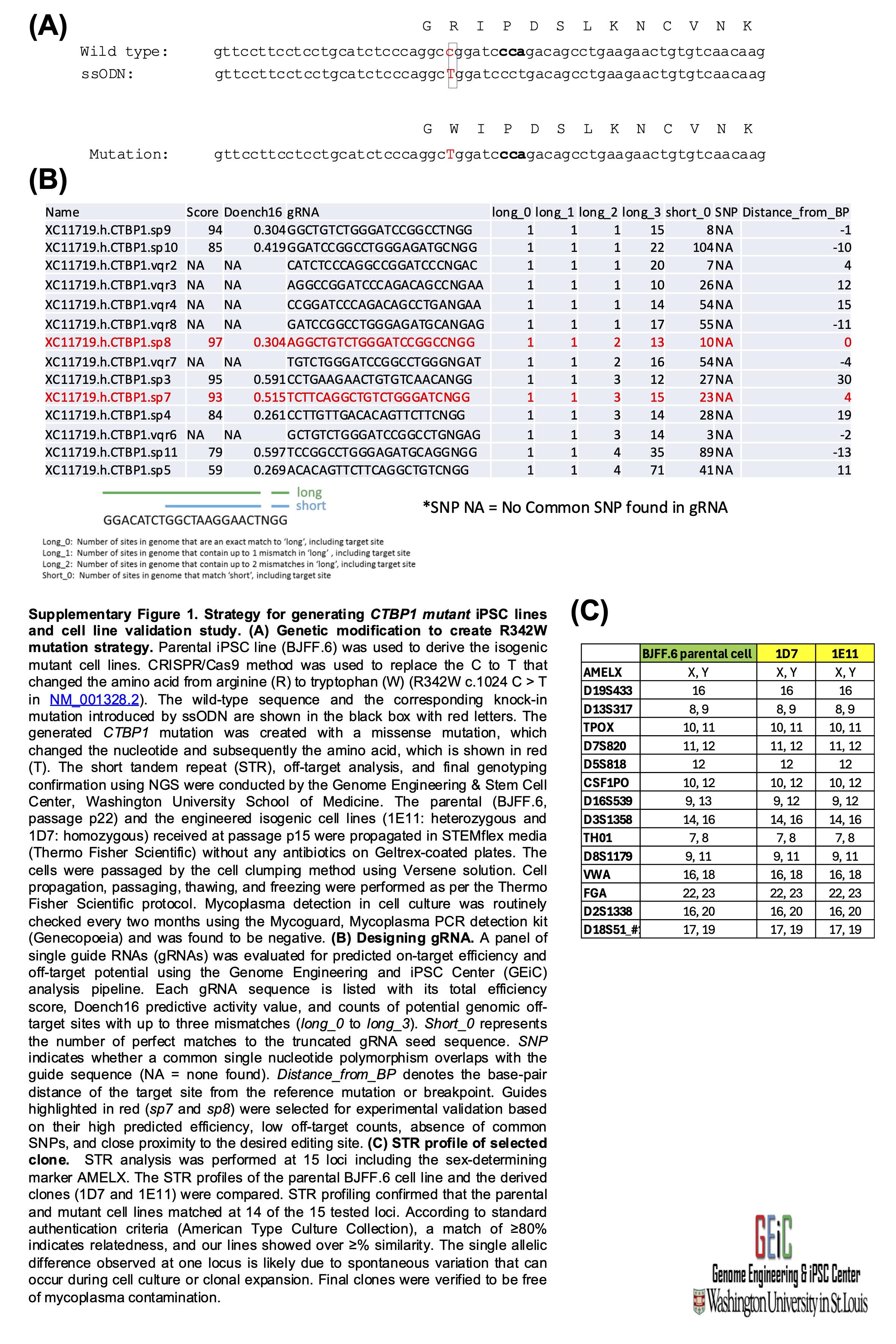

Supplement: Supplementary file 1 [file Data_Sheet_1.zip › High Resolution Supplementary/Supplementary Figure 1.jpg]

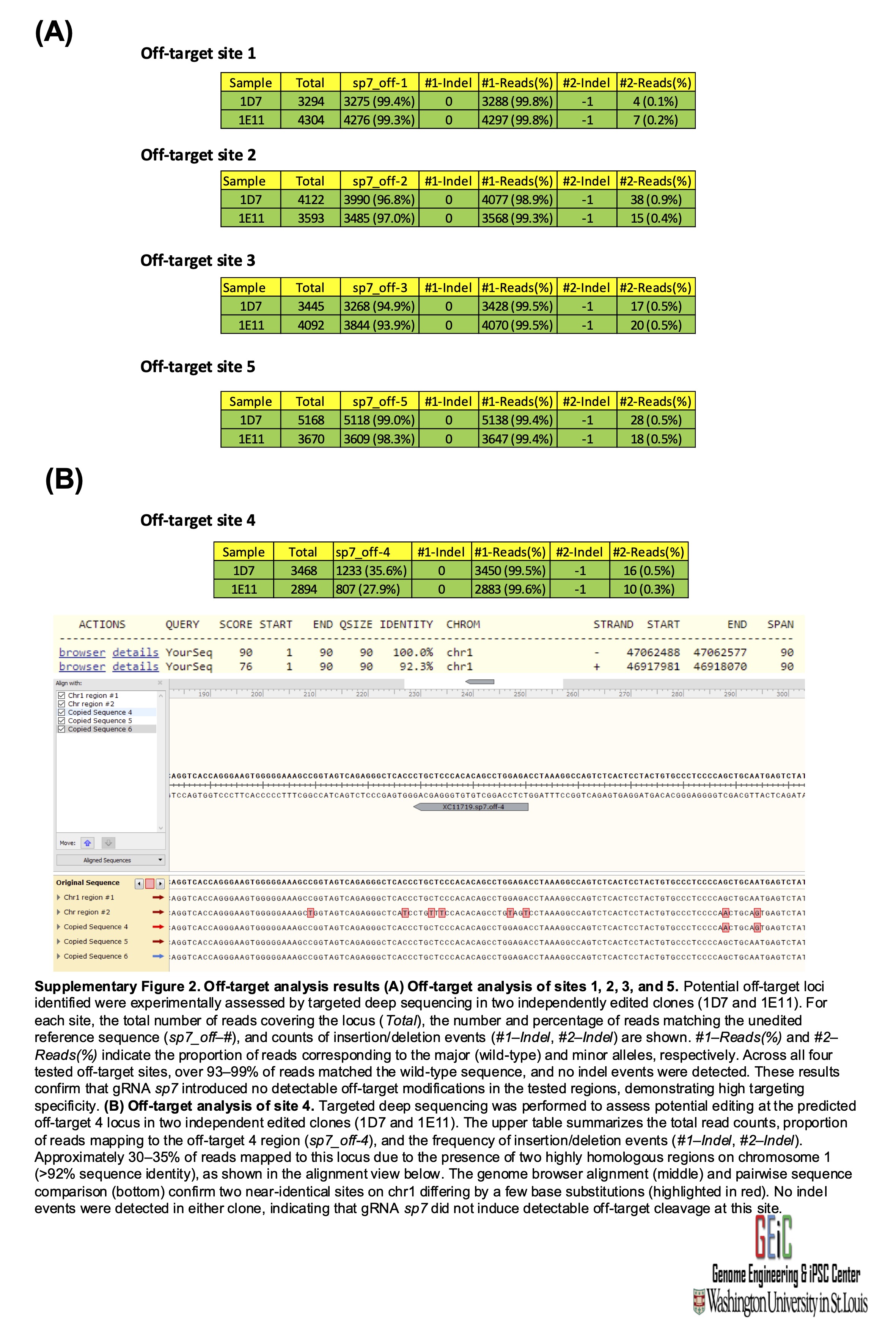

Supplement: Supplementary file 1 [file Data_Sheet_1.zip › High Resolution Supplementary/Supplementary Figure 2.jpg]

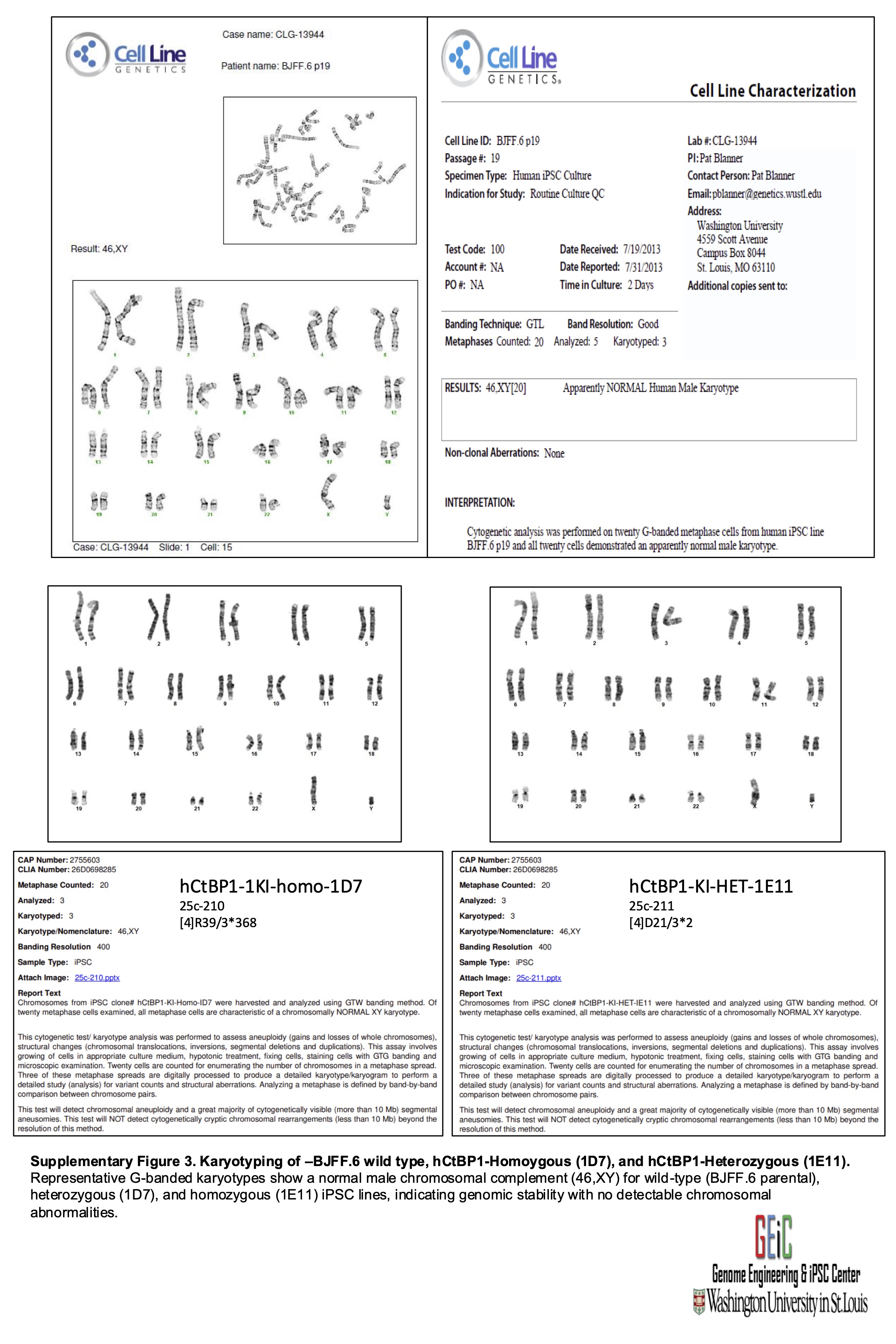

Supplement: Supplementary file 1 [file Data_Sheet_1.zip › High Resolution Supplementary/Supplementary Figure 3.jpg]

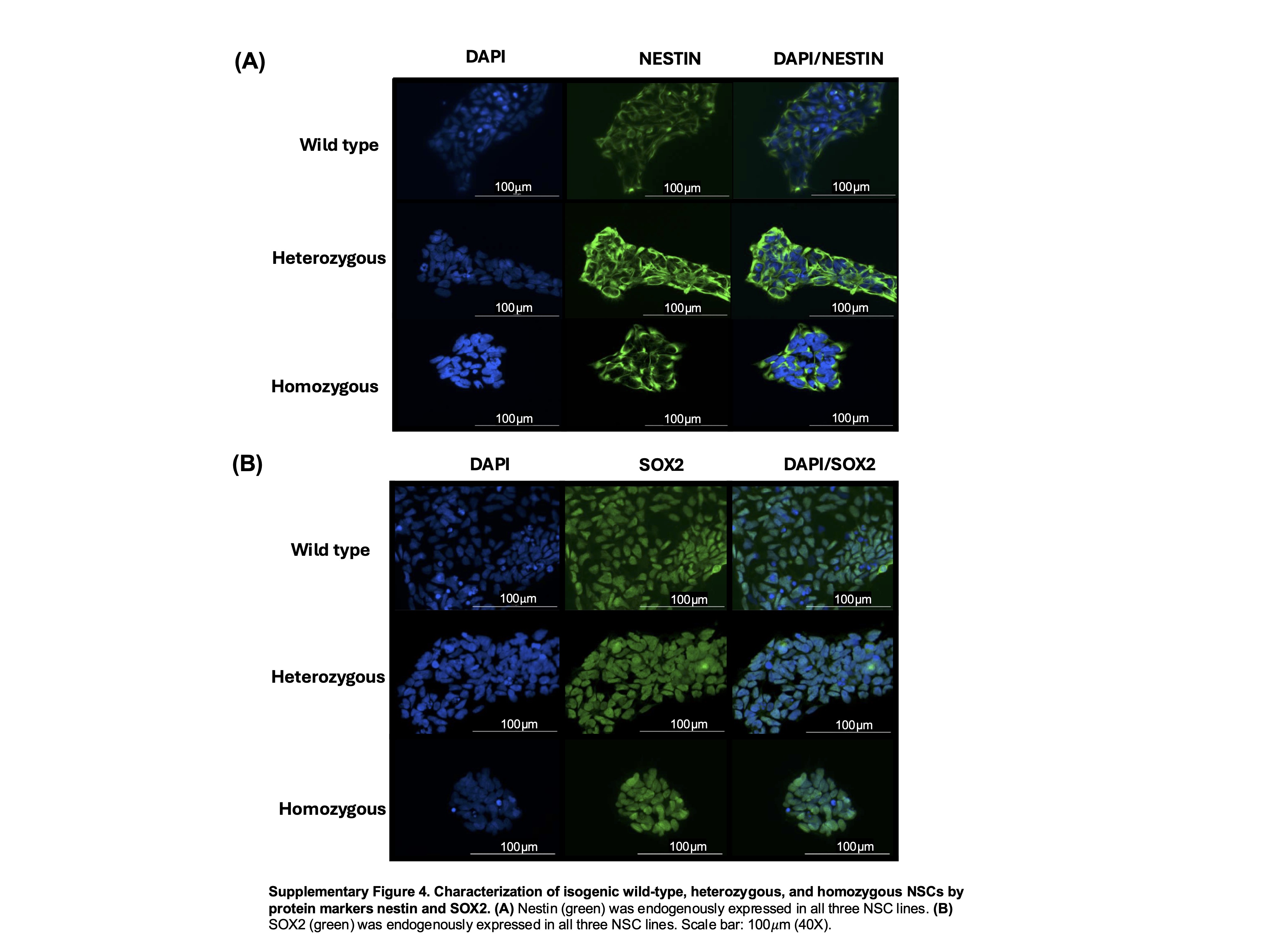

Supplement: Supplementary file 1 [file Data_Sheet_1.zip › High Resolution Supplementary/Supplementary Figure 4_NSC-immunocytochemistry.jpg]

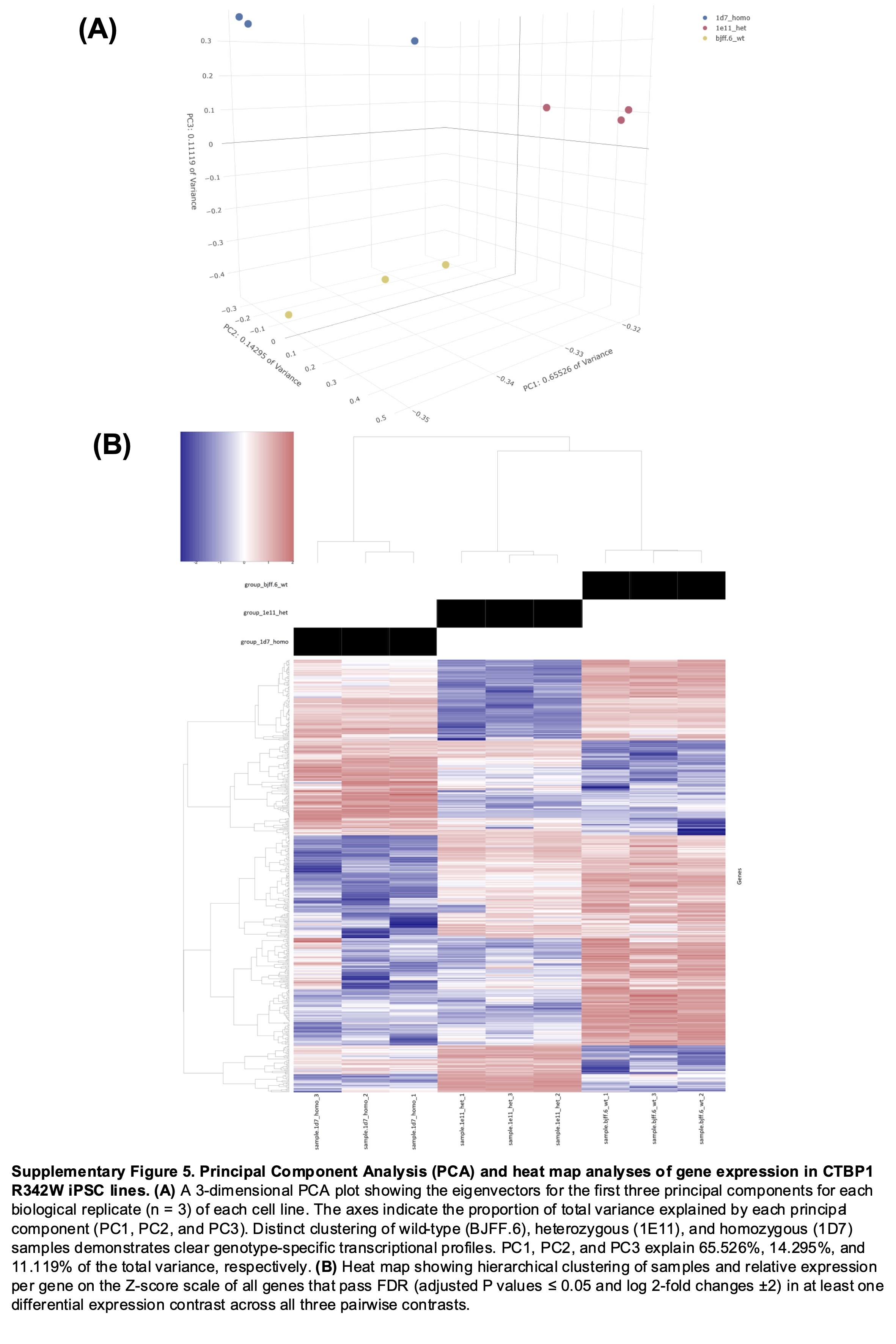

Supplement: Supplementary file 1 [file Data_Sheet_1.zip › High Resolution Supplementary/Supplementary Figure 5.jpg]

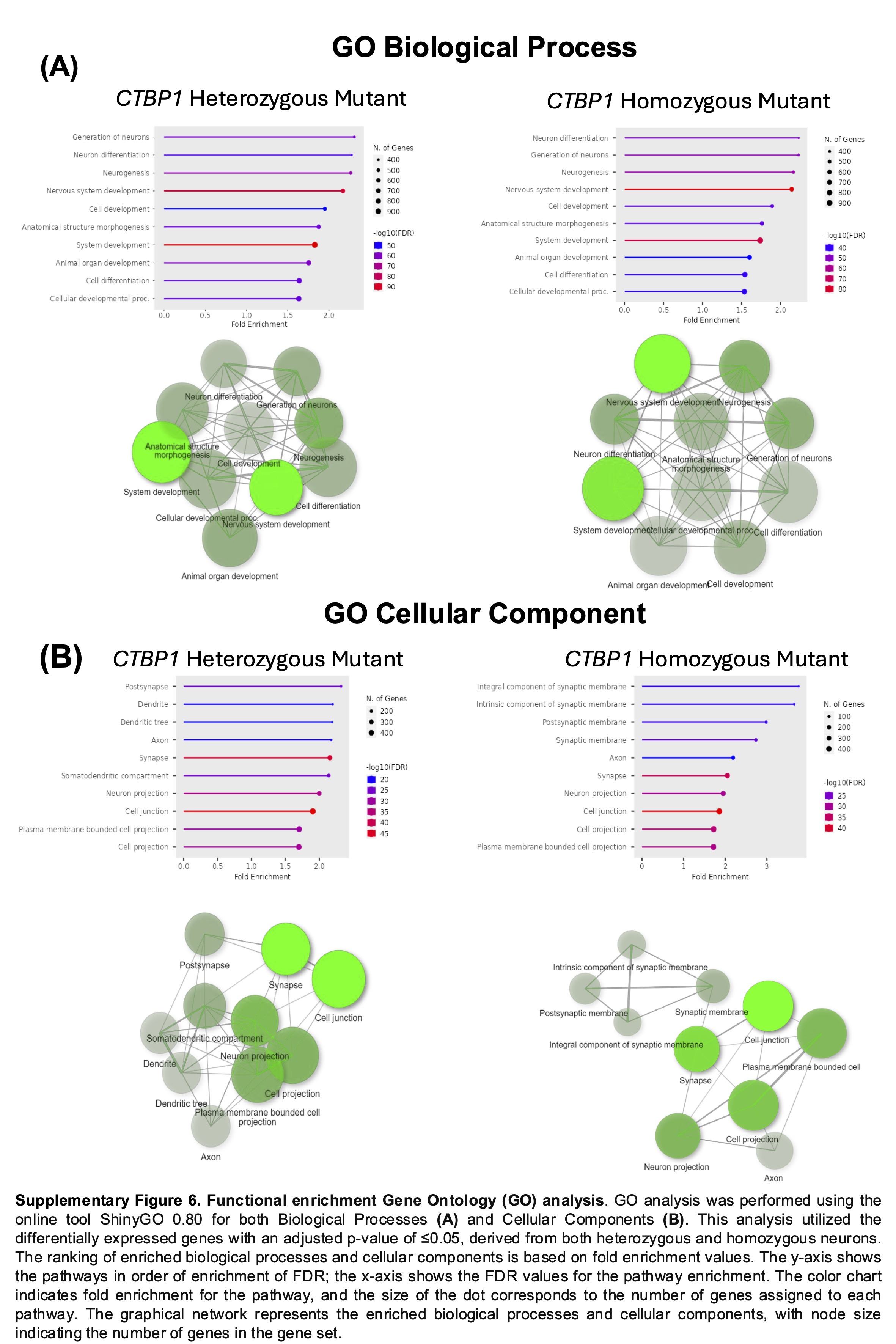

Supplement: Supplementary file 1 [file Data_Sheet_1.zip › High Resolution Supplementary/Supplementary Figure 6 Functional_gene_Enrichment.jpg]

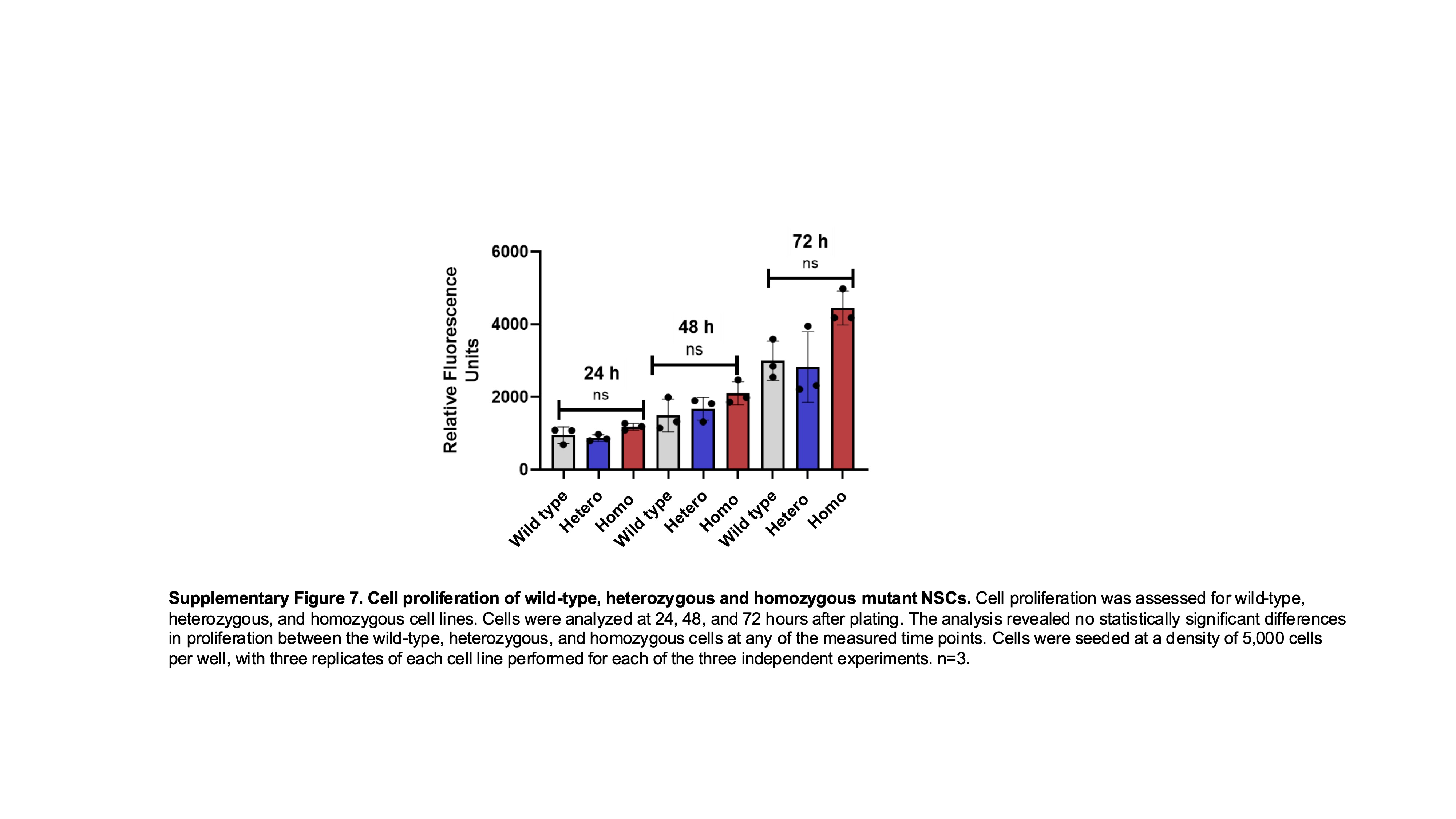

Supplement: Supplementary file 1 [file Data_Sheet_1.zip › High Resolution Supplementary/Supplementary Figure_7 Cell Proliferation.jpg]

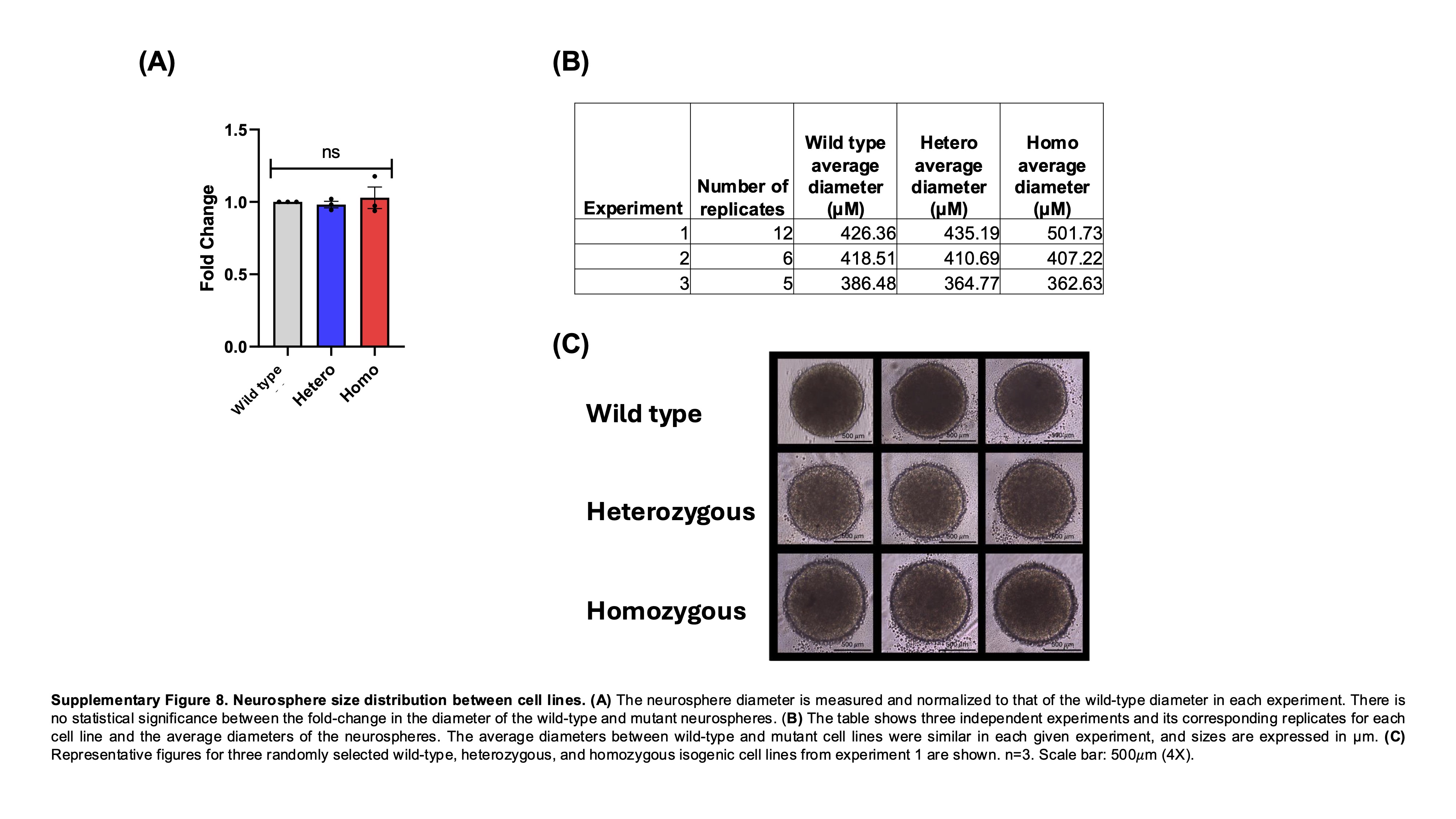

Supplement: Supplementary file 1 [file Data_Sheet_1.zip › High Resolution Supplementary/Supplementary Figure_8. Neurosphere_supplement.jpg]
